# Synthesis, Antiprotozoal Activity, and Cheminformatic Analysis of 2-Phenyl-2*H*-Indazole Derivatives

**DOI:** 10.3390/molecules26082145

**Published:** 2021-04-08

**Authors:** Karen Rodríguez-Villar, Lilián Yépez-Mulia, Miguel Cortés-Gines, Jacobo David Aguilera-Perdomo, Edgar A. Quintana-Salazar, Kevin Samael Olascoaga Del Angel, Francisco Cortés-Benítez, Juan Francisco Palacios-Espinosa, Olivia Soria-Arteche, Jaime Pérez-Villanueva

**Affiliations:** 1 Doctorado en Ciencias Biológicas y de la Salud, Universidad Autónoma Metropolitana (UAM), Ciudad de México 04960, Mexico; qkarenrodv@hotmail.com; 2 Unidad de Investigación Médica en Enfermedades Infecciosas y Parasitarias, UMAE Hospital de Pediatría, Centro Médico Siglo XXI, Instituto Mexicano del Seguro Social, Ciudad de México 06720, Mexico; lilianyepez@yahoo.com; 3 Departamento de Sistemas Biológicos, División de Ciencias Biológicas y de la Salud, Universidad Autónoma Metropolitana-Xochimilco (UAM-X), Ciudad de México 04960, Mexico; mcortes@pharmometrica.com.mx (M.C.-G.); jacoboaguilera.96@gmail.com (J.D.A.-P.); edgarqsl2811@gmail.com (E.A.Q.-S.); jcortesb@correo.xoc.uam.mx (F.C.-B.); jpalacios@correo.xoc.uam.mx (J.F.P.-E.); soriao@correo.xoc.uam.mx (O.S.-A.); 4 Doctorado en Biología Experimental, Universidad Autónoma Metropolitana (UAM), Ciudad de México 04960, Mexico; olaskuaga@gmail.com

**Keywords:** *Entamoeba histolytica*, *Giardia intestinalis*, *Trichomonas vaginalis*, indazole, structure-activity relationships

## Abstract

Indazole is an important scaffold in medicinal chemistry. At present, the progress on synthetic methodologies has allowed the preparation of several new indazole derivatives with interesting pharmacological properties. Particularly, the antiprotozoal activity of indazole derivatives have been recently reported. Herein, a series of 22 indazole derivatives was synthesized and studied as antiprotozoals. The 2-phenyl-2*H*-indazole scaffold was accessed by a one-pot procedure, which includes a combination of ultrasound synthesis under neat conditions as well as Cadogan’s cyclization. Moreover, some compounds were derivatized to have an appropriate set to provide structure-activity relationships (SAR) information. Whereas the antiprotozoal activity of six of these compounds against *E. histolytica*, *G. intestinalis*, and *T. vaginalis* had been previously reported, the activity of the additional 16 compounds was evaluated against these same protozoa. The biological assays revealed structural features that favor the antiprotozoal activity against the three protozoans tested, e.g., electron withdrawing groups at the 2-phenyl ring. It is important to mention that the indazole derivatives possess strong antiprotozoal activity and are also characterized by a continuous SAR.

## 1. Introduction

Indazole is an important scaffold in medicinal chemistry. So far, a few drugs containing the indazole moiety are currently available for clinical use; for instance, the antiemetic granisetron, the nonsteroidal anti-inflammatory drugs benzydamine and bendazac, the anticancer agents pazopanib, axitinib, niraparib and entrectinib. From these, niraparib and entrectinib were recently approved in 2017 and 2019 respectively [1,2]. At present, the progress on synthetic methodologies has allowed the preparation of several new indazole derivatives with interesting pharmacological properties [3,4,5,6]. Particularly, the antiprotozoal activity of indazole derivatives against *Entamoeba histolytica* [7], *Giardia intestinalis* and *Trichomonas vaginalis* have been recently reported [8,9].

*E. histolytica* and *G. intestinalis* are intestinal protozoa, which causes amebiasis and giardiasis, respectively. Worldwide 50 million amebiasis cases and 100,000 deaths annually are estimated [10], whereas 280 million people are affected by *G. intestinalis* [11]. Both diseases have high impact in the public health, causing diarrhea, which is a major factor in morbidity and mortality, affecting mainly infant population [12]. On the other hand, trichomoniasis, caused by *T. vaginalis* is the most common non-viral sexually transmitted disease [13]. According to the World Health Organization, more than 156 million new cases are estimated annually worldwide [14]. Trichomoniasis can be the cause of cervicitis, urethritis, vaginitis, and genital ulceration; additionally, it has been associated with preterm labor, low-birth weight, sterility, cervical cancer, and a predisposition to HIV infection [14,15,16]. Although some chemotherapeutic agents are available to treat amebiasis, giardiasis and trichomoniasis, e.g., nitroderivatives such as metronidazole [13,16,17], the increased drug resistance [13,16,17,18,19], and absence of new approved drugs with alternative mechanism of action, lead us to search new antiprotozoal compounds, particularly based on the indazole scaffold. Herein, 22 indazole derivatives were synthesized employing a practical one-pot process. Whereas six of these compounds have been previously reported to have activity against *E. histolytica*, *G. intestinalis*, and *T. vaginalis*, in this study, the activity of the additional 16 compounds against these three parasites was evaluated. Moreover, a SAR analysis and activity landscape studies were performed to highlight relevant structural features for the biological activity and to identify the SAR nature of the compounds studied.

## 2. Results and Discussion

### 2.1. Synthesis of 2-Phenyl-2H-Indazole Derivatives

**Scheme 1:**
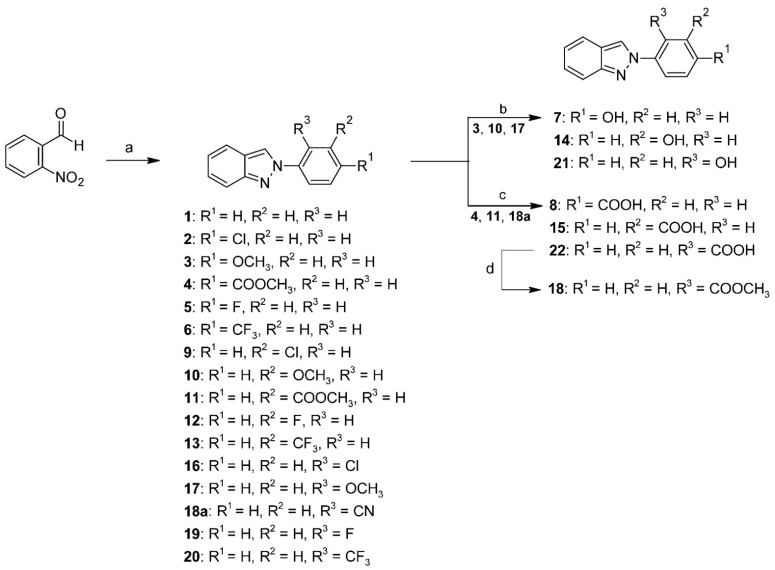
Synthesis of the 2-phenylindazole derivatives. Reagents and conditions: (**a**) 1. R^1^C_6_H_4_NH_2_, ultrasound, 40 °C, neat, 2. P(OEt)_3_, reflux; (**b**) BBr_3_, CH_2_Cl_2_, 0 °C; (**c**) 1. NaOH, H_2_O, reflux, 2. HCl; (**d**) HCl, MeOH, MW.

The synthetic route for the synthesis of 2-phenyl-2*H*-indazole derivatives is shown in Scheme 1. For this purpose, a new practical modification of the Cadogan’s method using an ultrasound assisted one-pot synthesis was employed. This method uses a combination of ultrasound synthesis as previously described by Crawford under neat conditions as well as the Cadogan’s cyclization by refluxing the Schiff base with triethyl phosphite [20,21]. A comparison of the employed one-pot procedure and a previously reported methodology for representative examples is displayed in Table 1. Noteworthy, the one-pot method leads to similar or better yields than our previous report [8]. Moreover, it is important to mention that our method avoids one purification step, save solvents, and reduce the work up time. To expand the scope of the method, the number of compounds available for biological assays, and the diversity of substituents for SAR analysis, derivatives with chlorine, methoxy, methoxycarbonyl, fluorine and trifluoromethyl groups at the 2- or 3-positions of the phenyl substituent were also synthesized. It is to be noted that compound **18**, having a 2-(methoxycarbonyl)phenyl substituent, was not obtained by employing the described method, instead, **18a** having a 2-cyanophenyl was synthesized as alternative intermediary to obtain **18**; however a slight method modification was also needed to achieve the compound **18a** (*vide infra*). Since compounds substituted with OH and COOH are important from the medicinal chemistry point of view due to the fact that it forms key protein-ligand interactions (e.g., hydrogen bonds and salt bridges) [22], derivatives having hydroxyl (**7**, **14** and **21**) and carboxyl (**8**, **15** and **22**) groups were synthesized by hydrolysis of precursors **3**, **10**, **17**, **4**, **11** and **18a**. All synthesized compounds were characterized by ^1^H NMR and ^13^C NMR spectra and the new structures were also characterized by mass spectrometry. The nuclear magnetic resonance spectra of all compounds can be found in Figures S1–S23 in the Supplementary Materials.

### 2.2. Antiprotozoal Activity

**Figure 1:**
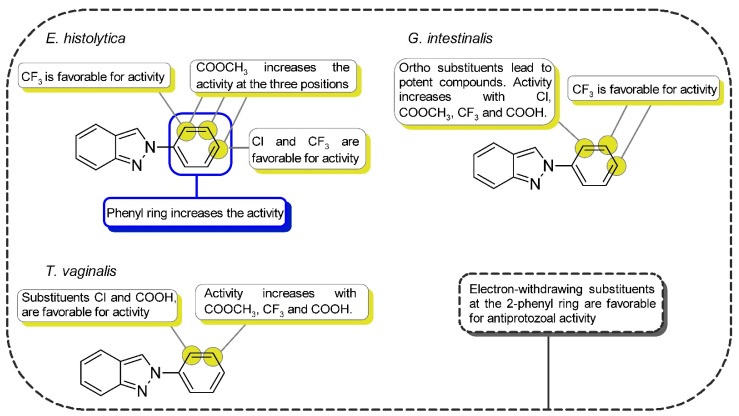
Principally SAR features derived from the 2-phenyl-2*H*-indazole derivatives tested.

Compounds **5**, **6**, **9**–**22** were tested in vitro against *E. histolytica*, *G. intestinalis*, and *T. vaginalis*. It is important to mention that the activity of compounds **1**–**4**, **7** and **8** against these three protozoa were previously reported by our group [8], however, the values were included in Table 2 in order to support the SAR analysis. Against *E. histolytica*, the first interesting finding was that all tested compounds were more potent than 1*H*-indazole reported by López-Vallejo et al. (IC_50_ = 0.740 µM) [7]. It is important to note that biological assays were performed using the same strain, under the same conditions as well as the same laboratory. The results suggest that 2-phenyl substitution is important for the antiprotozoal activity, since this modification increased 9-fold the activity. The best potency was found for 2-phenyl-2*H*-indazole derivatives substituted with methoxycarbonyl (**4**, **11** and **18**), 4-chlorophenyl (**2**) and 2-(trifluoromethyl)phenyl (**20**) (IC_50_ < 0.050 µM). On the other hand, the results for the assays performed against *G. intestinalis* showed that the derivatives substituted with 2-chlorophenyl (**16**), 2-(methoxycarbonyl)phenyl (**18**), 2-(trifluoromethyl)phenyl (**20**) and 2-carboxyphenyl (**22**) displayed the best activity (IC_50_ < 0.050 µM). Moreover, 4- and 3-(trifluoromethyl)phenyl derivatives (**6** and **13** respectively) showed favorable effect on the giardicidal activity. It is worth to emphasize that compounds were slightly more potent against *E. histolytica* and *G. intestinalis* than *T. vaginalis*. Hence, against *T. vaginalis*, the best activity (IC_50_ < 0.070 µM) was found for the derivatives substituted with 3-(methoxycarbonyl)phenyl (**11**), 3-(trifluoromethyl)phenyl (**13**) and 3-carboxyphenyl (**15**), as well as the 2-chlorophenyl (**16**) and 2-carboxyphenyl (**22**). The IC_50_ values and SAR data displayed in Figure 1, indicate that electron-withdrawing substituents attached to the 2-phenyl ring are favorable for the antiprotozoal activity against the three evaluated protozoa.

### 2.3. Cheminformatic Analysis

**Figure 2:**
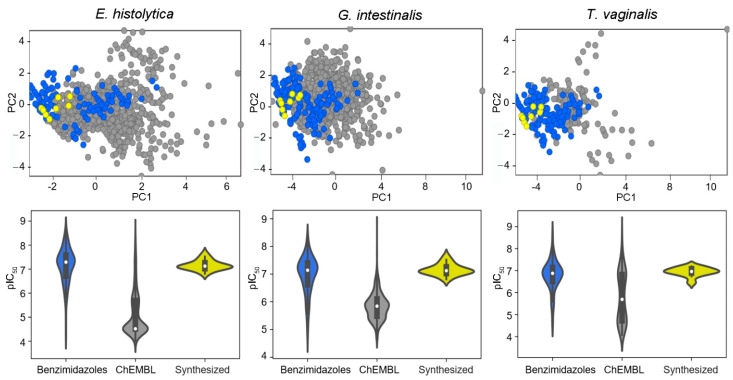
Property space and activity range for 2-phenyl-2*H*-indazole derivatives (**1**–**22**) synthesized in this work (yellow), a collection of antiprotozoal 1*H*-benzimidazole derivatives (blue) and ChEMBL collection of antiprotozoals (gray). The two principal components taken together account for 75.34, 72.44 and 75.71% of the variance for *E. histolytica*, *G. intestinalis*, and *T. vaginalis*, respectively.

Compounds **1**–**22** were also analyzed employing cheminformatic tools and compared against antiprotozoal databases of benzimidazole derivatives [7,23,24,25,26,27,28,29,30,31,32,33,34,35,36] and ChEMBL compounds [37], which have also reported activities against *E. histolytica*, *G. intestinalis* and *T. vaginalis*. It is worthwhile to mention that benzimidazole derivatives compose an important reference database since a high number of compounds were tested under the same method and conditions. These benzimidazole derivatives have been the reference database for several cheminformatic and QSAR studies as antiprotozoals [31,34,38,39,40]; and the data available has been expanded during the last years. Figure 2 shows a representation of the property space and the activity distribution of the 22 synthesized indazole derivatives as compared to benzimidazole derivatives and ChEMBL collection for each analyzed parasite [41]. Although, 2-phenyl-2*H*-indazole derivatives are localized in a very focalized space as compared to benzimidazole and ChEMBL compounds, their activity values are similar to the majority of benzimidazoles, but higher than ChEMBL compounds in most cases, as is visually depicted as violin plots in Figure 2. Although the number and diversity of indazole derivatives synthesized and tested herein are still low, it is of our interest to expand the database of indazole derivatives as well as their activity landscape to search for more potent compounds.

**Figure 3:**
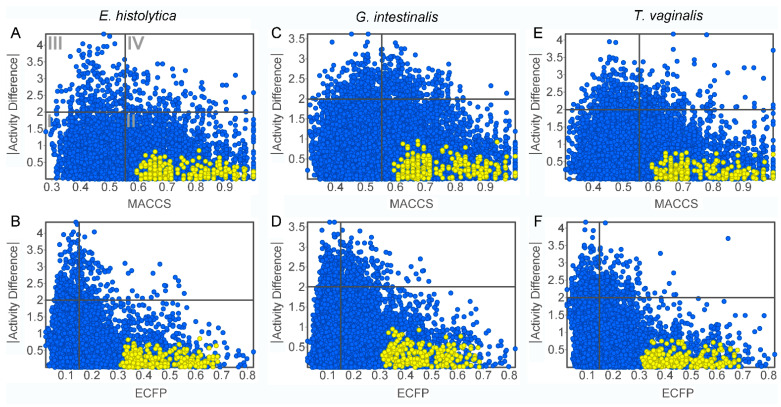
Structure-Activity Similarity Maps using Molecular ACCess System keys (MACCS) and Extended Connectivity Fingerprint (ECFP) divided by thresholds in structure similarity and activity differences in regions I–IV. Each data point indicates a pairwise comparison of the 22 synthesized 2-phenyl-2*H*-indazole derivatives (231 datapoints in yellow, **A**–**F**) and 111 benzimidazoles tested against *E. histolytica* (6105 datapoints in blue, **A**,**B**), 159 benzimidazoles tested against *G. intestinalis* (12,561 datapoints in blue, **C**,**D**), and 134 benzimidazoles tested against *T. vaginalis* (8911 datapoints in blue, **E**,**F**).

**Figure 4:**
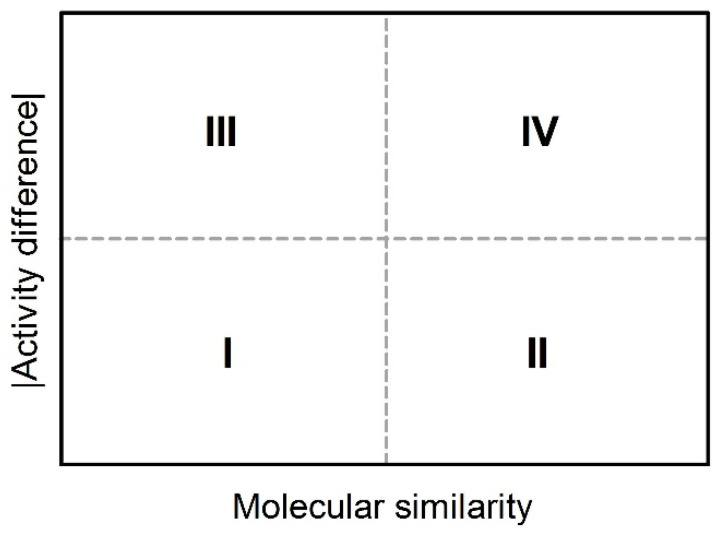
General form of the structure–activity similarity (SAS) maps showing four major regions.

To visualize the activity landscape of the synthesized indazole derivatives as compared to the benzimidazole reference database, chemotype-based Structure-Activity Similarity (SAS) maps were generated (Figure 3) [42]. A general form of the SAS map is presented in Figure 4. SAS maps are divided in four regions: region I is associated with scaffold hopping (or side chain hopping), region II denotes smooth SAR and region IV indicates discontinuous SAR and activity cliffs. The structure similarity threshold was established by the median similarity of the pairwise comparisons plotted in each map and depends on the database and the fingerprint employed, whereas the activity similarity threshold was established on two units in activity difference (100-fold difference in potency). Since Similarity-Property principle establish that similar compounds have similar properties (e.g., biological activity), all indazole derivatives fall in such definition [43]. Different SAR classifications have been previously defined as continuous (smooth), heterogeneous and discontinuous (rough) SAR [43,44]. Moreover, the implications in medicinal chemistry, disadvantages or opportunities implied on different SAR classification has been widely discussed [44,45]. Therefore, the 22 indazole derivatives studied are characterized by a smooth or continuous SAR (high similarity and low activity differences; region II) as compared to the benzimidazole derivatives database where a heterogeneous SAR can be observed [38,39]. Therefore, the antiprotozoal indazole database should be expanded by including more diverse substituted derivatives at different positions to carry out QSAR studies and lead optimization.

## 3. Materials and Methods

### 3.1. Chemicals and Instruments

All chemicals and starting materials were obtained from Sigma-Aldrich (Sigma-Aldrich, Toluca, MEX, Mexico). Reactions were monitored by TLC on 0.2 mm percolated silica gel 60 F_254_ plates (Merck, Darmstadt, Germany) and visualized by irradiation with a UV lamp (Cole-Parmer, Vernon Hills, IL, USA). Silica gel 60, 70–230 mesh, was used for column chromatography (Macherey-Nagel, Düren, Germany). Ultrasound assisted synthesis were carried out employing a Cole-Parmer ultrasonic bath model 8891 (Cole-Parmer, Vernon Hills, IL, USA), operating at 42 kHz. Melting points were determined in open capillary tubes with a Büchi M-565 melting point apparatus (Büchi, Flawil, Switzerland) and are uncorrected. Microwave-assisted reactions were carried out in a monomodal reactor (Anton-Paar Monowave 300) equipped with a hydraulic pressure sensing device and an infrared temperature-sensor (Anton-Paar, Graz, Austria). ^1^H NMR and ^13^C NMR were measured with an Agilent DD2 spectrometer (Agilent, Santa Clara, CA, USA), operating at 600 MHz and 151 MHz for ^1^H and ^13^C, respectively. Chemical shifts are given in parts per million relatives to tetramethylsilane (Me_4_Si, δ = 0); *J* values are given in Hz. Splitting patterns are expressed as follow: s, singlet; d, doublet; t, triplet; q, quartet; dd, doublet of doublet; td, triplet of doublet; ddd, doublet doublet of doublet; dq, doublet of quartets; m, multiplet; bs, broad singlet. High resolution Mass spectra were recorded on a Bruker ESI/APCI-TOF, MicroTOF-II-Focus spectrometer (Bruker, Billerica, MA, USA) by electrospray ionization (ESI); whereas low resolution Mass spectra were recorded on a Waters Xevo TQ-MS spectrometer (Waters, Milford, MA, USA) by electron impact (EI). All compounds were named using the automatic name generator tool from the ChemDraw Professional 16.0.1.4 software (PerkinElmer, Waltham, MA, USA), according to IUPAC rules.

### 3.2. Chemistry

#### 3.2.1. General Procedure for the One-Pot Synthesis of 2-Phenyl-2*H*-Indazole Derivatives **1**–**6**, **9**–**13**, **16**, **17**, **19** and **20**

A 50 mL heavy wall glass Schlenk reaction tube was charged with 2-nitrobenzaldehyde (1.654 mmol) and aniline or the substituted aniline (1.654 mmol). The reaction mixture was ultrasonicated for two hours at 40 °C. The condensed water under the tube walls was removed. Afterwards, triethyl phosphite (5 mmol) was added and heated to 150 °C under nitrogen atmosphere for two hours. The phosphite excess was oxidized with 20 mL of 5% hydrogen peroxide solution. The product was extracted with ethyl acetate (20 mL × 3) and the organic layer was washed with brine (20 mL) and dried with anhydrous sodium sulfate and finally concentrated under vacuum distillation. The evaporation residue was purified by using column chromatography with hexane–ethyl acetate 98:2 (compound **19**), 95:5 (compounds, **9**–**13, 16**, **17**), 90:10 (compounds **1**–**3**, **6**, **20**), and 80:20 (compounds **4**, **5**).

*2-Phenyl-2H-Indazole* (**1**), White solid, 92% yield, m.p.: 81–82 °C. The spectroscopic data agree with the previously reported data [8,46]. ^1^H NMR (600 MHz, CDCl_3_) δ 8.41 (d, *J* = 0.9 Hz, 1H), 7.91–789 (m, 2H), 7.79 (dd, *J* = 8.8, 0.9 Hz, 1H), 7.71 (dt, *J* = 8.5, 1.0 Hz, 1H), 7.54–7.49 (m, 2H), 7.41–7.37 (m, 1H), 7.32 (ddd, *J* = 8.8, 6.6, 1.0 Hz, 1H), 7.11 (ddd, *J* = 8.4, 6.6, 0.7 Hz, 1H); ^13^C NMR (151 MHz, CDCl_3_) δ 149.79, 140.54, 129.56, 127.90, 126.83, 122.77, 122.46, 121.00, 120.41, 120.38, 117.95.

*2-(4-Chlorophenyl)-2H-Indazole* (**2**), White solid, 62% yield, m.p.: 143–146 °C. The spectroscopic agree with the previously reported data [8,47]. ^1^H NMR (600 MHz, CDCl_3_) δ 8.37 (d, *J* = 1.0 Hz, 1H), 7.87–7.83 (m, 2H), 7.77 (dq, *J* = 8.8, 0.9 Hz, 1H), 7.69 (dt, *J* = 8.5, 1.0 Hz, 1H), 7.51–7.47 (m, 2H), 7.33 (ddd, *J* = 8.8, 6.6, 1.1 Hz, 1H), 7.12 (ddd, *J* = 8.5, 6.6, 0.8 Hz, 1H); ^13^C NMR (151 MHz, CDCl_3_) δ 150.05, 139.19, 133.72, 129.83, 127.26, 123.03, 122.87, 122.17, 120.50, 120.42, 118.07.

*2-(4-Methoxyphenyl)-2H-Indazole* (**3**), Beige solid, 57% yield, m.p.: 133–136 °C. The spectroscopic data agree with the previously reported data [8,48]. ^1^H NMR (600 MHz, CDCl_3_) δ 8.31 (d, *J* = 0.9 Hz, 1H), 7.83–7.76 (m, 3H), 7.70 (dt, *J* = 8.4, 1.0 Hz, 1H), 7.32 (ddd, *J* = 8.7, 6.6, 1.0 Hz, 1H), 7.11 (ddd, *J* = 8.4, 6.6, 0.8 Hz, 1H), 7.06–7.00 (m, 2H), 3.86 (s, 3H); ^13^C NMR (151 MHz, CDCl_3_) δ 159.42, 149.71, 134.25, 126.66, 122.83, 122.55, 122.35, 120.43, 120.38, 117.90, 114.77, 55.73.

*Methyl 4-(2H-Indazol-2-yl)benzoate* (**4**), White solid, 39% yield, m.p.: 186–187 °C. The spectroscopic data agree with the previously reported data [8,48]. ^1^H NMR (600 MHz, CDCl_3_) δ 8.47 (d, *J* = 0.7 Hz, 1H), 8.22–8.18 (m, 2H), 8.02–7.99 (m, 2H), 7.77 (dd, *J* = 8.8, 0.8 Hz, 1H), 7.69 (d, *J* = 8.5 Hz, 1H), 7.33 (ddd, *J* = 8.8, 6.6, 1.0 Hz, 1H), 7.14–7.10 (m, 1H), 3.95 (s, 3H); ^13^C NMR (151 MHz, CDCl_3_) δ 166.32, 150.32, 143.77, 131.30, 129.40, 127.58, 123.14, 123.12, 120.60, 120.39, 118.20, 52.46.

*2-(4-Fluorophenyl)-2H-Indazole* (**5**), White solid, 71% yield, m.p.: 110–111 °C. The spectroscopic data agree with the previously reported data [49]. ^1^H NMR (600 MHz, CDCl_3_) δ 8.34 (d, *J* = 0.9 Hz, 1H), 7.88–7.84 (m, 2H), 7.78 (dq, *J* = 8.8, 1.0 Hz, 1H), 7.70 (dt, *J* = 8.5, 1.0 Hz, 1H), 7.33 (ddd, *J* = 8.7, 6.6, 1.0 Hz, 1H), 7.23–7.19 (m, 2H), 7.12 (ddd, *J* = 8.5, 6.6, 0.8 Hz, 1H). ^13^C NMR (151 MHz, CDCl_3_) δ 162.05 (d, *J* = 248.1 Hz), 149.83 (s), 136.88 (d, *J* = 2.7 Hz), 126.94 (s), 122.84 (s), 122.77 (d, *J* = 8.6 Hz), 122.59 (s), 120.48 (s), 120.34 (s), 117.89 (s), 116.45 (d, *J* = 22.8 Hz).

*2-[4-(Trifluoromethyl)phenyl]-2H-Indazole* (**6**), Pale pink solid, 43% yield, m.p.: 188–190 °C. The spectroscopic data agree with the previously reported data [47]. ^1^H NMR (600 MHz, CDCl_3_) δ 8.46 (d, *J* = 0.9 Hz, 1H), 8.06 (d, *J* = 8.5 Hz, 2H), 7.81–7.76 (m, 3H), 7.70 (dt, *J* = 8.5, 1.0 Hz, 1H), 7.34 (ddd, *J* = 8.8, 6.6, 1.0 Hz, 1H), 7.13 (ddd, *J* = 8.4, 6.6, 0.5 Hz, 1H). ^13^C NMR (600 MHz, CDCl_3_) δ 150.21, 142.95, 129.75 (q, *J* = 33.1 Hz), 127.52, 126.86 (q, *J* = 3.7 Hz), 123.80 (q, *J* = 272.0 Hz), 123.08, 123.04, 120.76, 120.47, 120.41, 118.09.

*2-(3-Chlorophenyl)-2H-Indazole* (**9**), Light pink solid, 53% yield, m.p.: 109–110 °C. The spectroscopic data agree with the previously reported data [48,50]. ^1^H NMR (600 MHz, CDCl_3_) δ 8.39 (d, *J* = 1.0 Hz, 1H), 7.98 (t, *J* = 2.1 Hz, 1H), 7.81–7.75 (m, 2H), 7.70 (dt, *J* = 8.5, 1.0 Hz, 1H), 7.45 (t, *J* = 8.1 Hz, 1H), 7.39–7.31 (m, 2H), 7.12 (ddd, *J* = 8.3, 6.6, 0.8 Hz, 1H); ^13^C NMR (151 MHz, CDCl_3_) δ 149.95, 141.45, 135.42, 130.57, 127.88, 127.25, 122.83, 121.24, 120.41, 120.39, 118.77, 117.99.

*2-(3-Methoxyphenyl)-2H-Indazole* (**10**), Beige solid, 45% yield, m.p.: 51–52 °C. The spectroscopic data agree with the previously reported data [50]. ^1^H NMR (600 MHz, CDCl_3_) δ 8.39 (d, *J* = 1.0 Hz, 1H), 7.79 (dq, *J* = 8.6, 0.8 Hz, 1H), 7.69 (dt, *J* = 8.5, 1.0 Hz, 1H), 7.52 (t, *J* = 2.2 Hz, 1H), 7.44–7.38 (m, 2H), 7.32 (ddd, *J* = 8.8, 6.6, 1.1 Hz, 1H), 7.11 (ddd, *J* = 8.3, 6.5, 0.8 Hz, 1H), 6.93 (ddd, *J* = 7.9, 2.5, 1.2 Hz, 1H), 3.90 (s, 3H); ^13^C NMR (151 MHz, CDCl_3_) δ 160.58, 149.72, 141.68, 130.27, 126.87, 122.71, 122.48, 120.57, 120.39, 117.94, 113.94, 112.94, 106.81, 55.61. 

*Methyl 3-(2H-Indazol-2-yl)benzoate* (**11**), Beige solid, 51% yield, m.p.: 103–104 °C. The spectroscopic data agree with the previously reported data [48]. ^1^H NMR (600 MHz, CDCl_3_) 8.55–8.53 (m, 1H), 8.48 (d, *J* = 0.9 Hz, 1H), 8.18 (ddd, *J* = 8.1, 2.3, 1.1 Hz, 1H), 8.08–8.05 (m, 1H), 7.79 (dq, *J* = 8.8, 0.9 Hz, 1H), 7.71 (dt, *J* = 8.5, 1.1 Hz, 1H), 7.61 (t, *J* = 7.9 Hz, 1H), 7.33 (ddd, *J* = 8.7, 6.6, 1.0 Hz, 1H), 7.12 (ddd, *J* = 8.5, 6.6, 0.9 Hz, 1H), 3.97 (s, 3H); ^13^C NMR (151 MHz, CDCl_3_) δ 166.10, 149.98, 140.67, 131.69, 129.79, 128.77, 127.18, 125.21, 122.93, 122.77, 121.51, 120.46, 120.44, 118.00, 52.47. 

*2-(3-Fluorophenyl)-2H-Indazole* (**12**), White solid, 51% yield, m.p.: 96–98 °C. The spectroscopic data agree with the previously reported data [48]. ^1^H NMR (600 MHz, CDCl_3_) δ 8.40 (m, *J* = 0.8 Hz, 1H), 7.79–7.76 (m, 1H), 7.73–7.67 (m, 3H), 7.51–7.46 (m, 1H), 7.33 (ddd, *J* = 8.8, 6.6, 0.9 Hz, 1H), 7.11 (m, 2H); ^13^C NMR (151 MHz, CDCl_3_) δ 163.22 (d, *J* = 247.4 Hz), 141.88 (d, *J* = 10.0 Hz), 130.88 (d, *J* = 9.1 Hz), 127.24 (s), 122.87 (s), 122.83 (s), 120.42 (s), 120.41 (s), 118.03 (s), 116.14 (d, *J* = 3.1 Hz), 114.71 (d, *J* = 21.2 Hz), 108.70 (d, *J* = 26.2 Hz).

*2-[3-(Trifluoromethyl)phenyl]-2H-Indazole* (**13**), White solid, 46% yield, m.p.: 73–75 °C. The spectroscopic data agree with the previously reported data [48]. ^1^H NMR (600 MHz, DMSO-*d*_6_) δ 9.29 (d, *J* = 0.9 Hz, 1H), 8.48–8.43 (m, 2H), 7.87–7.81 (m, 2H), 7.80 (dt, *J* = 8.5, 1.0 Hz, 1H), 7.74 (dq, *J* = 8.8, 0.9 Hz, 1H), 7.36 (ddd, *J* = 8.8, 6.5, 1.1 Hz, 1H), 7.14 (ddd, *J* = 8.5, 6.5, 0.8 Hz, 1H); ^13^C NMR (151 MHz, DMSO-*d*_6_) δ 149.64, 140.82, 131.55, 130.88 (q, *J* = 32.9 Hz), 127.84, 124.69 (q, *J* = 3.8 Hz), 124.18 (q, *J* = 272.6 Hz), 123.03, 122.96, 122.72, 121.52, 117.96, 117.14 (q, *J* = 3.9 Hz).

*2-(2-Chlorophenyl)-2H-Indazole* (**16**), White solid, 55% yield, m.p.: 60–61 °C. The spectroscopic data agree with the previously reported data [51]. ^1^H NMR (600 MHz, CDCl_3_) δ 8.34 (d, *J* = 1.0 Hz, 1H), 7.80 (dq, *J* = 8.6, 0.8 Hz, 1H), 7.74 (dt, *J* = 8.5, 1.0 Hz, 1H), 7.70–7.68 (m, 1H), 7.59–7.56 (m, 1H), 7.45–7.40 (m, 2H), 7.35 (ddd, *J* = 8.7, 6.6, 1.0 Hz, 1H), 7.14 (ddd, *J* = 8.4, 6.6, 0.7 Hz, 1H); ^13^C NMR (151 MHz, CDCl_3_) δ 149.40, 138.60, 130.67, 129.94, 128.97, 128.55, 127.68, 126.92, 125.20, 122.42, 121.99, 120.51, 117.91.

*2-(2-Methoxyphenyl)-2H-Indazole* (**17**), Light brown oil, 69% yield. The spectroscopic data agree with the previously reported data [50]. ^1^H NMR (600 MHz, CDCl_3_) δ 8.51 (d, *J* = 1.0 Hz, 1H), 7.87 (dd, *J* = 7.9, 1.7 Hz, 1H), 7.78 (dq, *J* = 8.7, 0.9 Hz, 1H), 7.72 (dt, *J* = 8.4, 1.0 Hz, 1H), 7.39 (ddd, *J* = 8.2, 7.5, 1.7 Hz, 1H), 7.31 (ddd, *J* = 8.7, 6.6, 1.1 Hz, 1H), 7.14–7.07 (m, 3H), 3.89 (s, 3H); ^13^C NMR (151 MHz, CDCl_3_) δ 151.89, 148.82, 129.95, 129.25, 126.63, 126.52, 125.51, 122.03, 121.84, 121.20, 120.52, 117.71, 112.36, 56.04.

*2-(2-Fluorophenyl)-2H-Indazole* (**19**), Yellow oil, 54% yield. The spectroscopic data agree with the previously reported data [50,52]. ^1^H NMR (600 MHz, CDCl_3_) δ 8.50 (dd, *J* = 2.8, 0.9 Hz, 1H), 8.08 (m, 1H), 7.79 (m, 1H), 7.72 (dt, *J* = 8.5, 0.9 Hz, 1H), 7.41–7.27 (m, 4H), 7.12 (ddd, *J* = 8.5, 6.6, 0.6 Hz, 1H). ^13^C NMR (151 MHz, CDCl_3_) δ 154.20 (d, *J* = 250.6 Hz), 129.12 (d, *J* = 7.9 Hz), 125.10 (d, *J* = 3.7 Hz), 124.66 (d, *J* = 10.0 Hz), 122.60 (d, *J* = 1.3 Hz), 117.00 (d, *J* = 20.6 Hz).

*2-[2-(Trifluoromethyl)phenyl]-2H-Indazole* (**20**), White solid, 26% yield, m.p.: 51–52 °C. The spectroscopic data agree with the previously reported data [51]. ^1^H NMR (600 MHz, CDCl_3_) δ 8.20 (s, 1H), 7.88–7.84 (m, 1H), 7.79 (dd, *J* = 8.8, 1.0 Hz, 1H), 7.75–7.70 (m, 2H), 7.66–7.62 (m, *J* = 9.7, 4.3 Hz, 2H), 7.36 (ddd, *J* = 8.8, 6.6, 1.0 Hz, 1H), 7.15 (ddd, *J* = 8.6, 6.6, 0.8 Hz, 1H); ^13^C NMR (151 MHz, CDCl_3_) δ 149.74, 139.11 (q, *J* = 1.6 Hz), 132.83, 129.67, 127.27 (q, *J* = 5.1 Hz), 127.09, 126.42 (q, *J* = 31.7 Hz), 125.87 (q, *J* = 1.9 Hz), 123.0275 (q, 273.9), 122.69, 122.24, 120.54, 118.05, 77.16.

#### 3.2.2. General Procedure for *O*-Demethylation

The methoxy-2-phenylindazole compounds (**3**, **10** or **17**) (4 mmol) were dissolved in dichloromethane (12 mL) and cooled to 0 °C under N_2_ atmosphere. Afterwards, boron tribromide (12 mmol, 12 mL of 1 M solution in dichloromethane) was added and the reaction mixture was stirred at room temperature overnight. The solvent was removed by evaporation and a saturated sodium bicarbonate solution was added. The resulting solid was filtered and dried under vacuum. The crude product was purified using a short column packed with silica gel and ethyl acetate-hexanes (6:4) as a mobile phase.

*4-(2H-Indazol-2-yl) phenol* (**7**), White solid, 64% yield, m.p.: 179–181 °C. The spectroscopic data agree with the previously reported data [8]. ^1^H NMR (600 MHz, DMSO-*d*_6_) δ 9.84 (s, 1H), 8.90 (d, *J* = 0.9 Hz, 1H), 7.90–7.83 (m, 2H), 7.74 (dt, *J* = 8.4, 1.0 Hz, 1H), 7.68 (dq, *J* = 8.8, 0.9 Hz, 1H), 7.28 (ddd, *J* = 8.7, 6.6, 1.1 Hz, 1H), 7.08 (ddd, *J* = 8.3, 6.6, 0.8 Hz, 1H), 6.97–6.92 (m, 2H); ^13^C NMR (151 MHz, DMSO-*d*_6_) δ 157.18, 148.56, 132.20, 126.19, 122.33, 121.84, 121.66, 120.87, 120.67, 117.21, 115.90.

*3-(2H-Indazol-2-yl)phenol* (**14**), Beige solid, 47% yield, m.p.: 131–132 °C. ^1^H NMR (600 MHz, DMSO-*d*_6_) δ 9.94 (s, 1H), 9.02 (d, *J* = 0.9 Hz, 1H), 7.75 (dt, *J* = 8.5, 1.0 Hz, 1H), 7.70 (dq, *J* = 8.8, 0.9 Hz, 1H), 7.51–7.48 (m, 2H), 7.36 (t, *J* = 8.0 Hz, 1H), 7.30 (ddd, *J* = 8.8, 6.6, 1.1 Hz, 1H), 7.09 (ddd, *J* = 8.5, 6.5, 0.9 Hz, 1H), 6.84 (ddd, *J* = 8.1, 2.4, 1.0 Hz, 1H); ^13^C NMR (151 MHz, DMSO-*d*_6_) δ 158.37, 148.77, 141.02, 130.47, 126.70, 122.35, 122.02, 121.51, 120.90, 117.44, 114.84, 110.67, 107.41. MS: *m*/*z* 211.08 ([M + H]^+^, 100%)

*2-(2H-Indazol-2-yl)phenol* (**21**), White solid, 85% yield, m.p.: 114–116 °C. The spectroscopic data agree with the previously reported data [52]. ^1^H NMR (600 MHz, CDCl_3_) δ 12.09 (s, 1H), 8.47 (d, *J* = 0.8 Hz, 1H), 7.75–7.71 (m, 2H), 7.58 (dd, *J* = 8.1, 1.5 Hz, 1H), 7.38 (ddd, *J* = 8.7, 6.6, 1.0 Hz, 1H), 7.29–7.25 (m, 1H), 7.19–7.15 (m, 2H), 6.97 (ddd, *J* = 8.2, 7.3, 1.4 Hz, 1H); ^13^C NMR (151 MHz, CDCl_3_) δ 150.48, 147.61, 129.08, 127.69, 124.84, 122.96, 121.32, 120.28, 120.21, 119.63, 119.43, 119.22, 116.82.

#### 3.2.3. General Procedure for the Hydrolysis of Ester Derivatives

The methyl ester compounds (**4** or **11**, 1.2 mmol) were dissolved in methanol (7.5 mL) and an aqueous solution of NaOH (3.6 mmol in 3 mL of water) was added. The reaction mixture was refluxed until the starting material was totally consumed. The mixture was cooled on ice and acidified to pH 1 with HCl to induce precipitation. The solid was filtered under vacuum and dried.

*4-(2H-Indazol-2-yl)benzoic Acid* (**8**), White solid, 96% yield, m.p.: 288–289 °C. The spectroscopic data agree with the previously reported data [8]. ^1^H NMR (600 MHz, DMSO-*d*_6_) δ 9.21 (s, 1H), 8.26–8.23 (m, 2H), 8.15–8.11 (m, 2H), 7.78 (dt, *J* = 8.5, 1.0 Hz, 1H), 7.72 (dd, *J* = 8.8, 0.9 Hz, 2H), 7.33 (ddd, *J* = 8.8, 6.5, 1.1 Hz, 1H), 7.12 (ddd, *J* = 8.5, 6.6, 0.8 Hz, 1H); ^13^C NMR (151 MHz, DMSO-*d*_6_) δ 166.54, 149.30, 142.91, 130.90, 129.74, 127.36, 122.62, 122.52, 122.13, 121.07, 119.94, 117.57.

*3-(2H-Indazol-2-yl)benzoic Acid* (**15**), White solid, 43% yield, m.p.: 215–217 °C. 1H NMR (600 MHz, acetone-*d*_6_) δ 9.01 (s, 1H), 8.78–8.69 (m, 1H), 8.36 (ddd, *J* = 8.1, 2.3, 1.0 Hz, 1H), 8.12–8.05 (m, 1H), 7.82–7.69 (m, *J* = 11.5, 9.0, 4.6 Hz, 3H), 7.33 (ddd, *J* = 8.7, 6.6, 0.9 Hz, 1H), 7.15–7.08 (m, 1H); ^13^C NMR (151 MHz, acetone-*d*_6_) δ 166.84, 150.61, 141.64, 133.09, 130.81, 129.46, 127.85, 125.33, 123.98, 123.28, 122.20, 122.05, 121.73, 118.63. MS: *m*/*z* 239.13 ([M + H]^+^, 100%).

#### 3.2.4. Synthesis of Compound **18** and **22**

*2-(2H-Indazol-2-yl)benzonitrile* (**18a**), Synthesized by a slight variation of the general method (Section 3.2.1). Briefly, the reaction mixture was ultrasonicated by four cycles of 1 h at 40 °C; after each 1h sonication step, the mixture was maintained under vacuum for 30 min at 30 mmHg and 40 °C. The cyclization with triethyl phosphite and the reaction workup were carried out as previously described (*vide supra*). The crude product was purified by using column chromatography with hexane–ethyl acetate 90:10. White solid, 32% yield, m.p.: 128–129 °C. The spectroscopic data agree with the previously reported data [50]. ^1^H NMR (600 MHz, CDCl_3_) δ 8.60 (d, *J* = 0.9 Hz, 1H), 7.97 (dd, *J* = 8.2, 1.1 Hz, 1H), 7.85 (dd, *J* = 7.8, 1.4 Hz, 1H), 7.82–7.76 (m, 2H), 7.54 (td, *J* = 7.7, 1.1 Hz, 1H), 7.36 (ddd, *J* = 8.8, 6.6, 1.1 Hz, 1H), 7.15 (ddd, *J* = 8.5, 6.6, 0.8 Hz, 1H); ^13^C NMR (151 MHz, CDCl_3_) δ 150.22, 142.36, 134.52, 134.04, 128.45, 127.68, 125.98, 123.42, 123.09, 122.84, 120.70, 118.03, 116.55, 106.82.

*2-(2H-Indazol-2-yl)benzoic Acid* (**22**), A 50 mL round bottom flask was charged with 2-(2*H*-indazol-2-yl)benzonitrile (2 mmol), sodium hydroxide (16 mmol) and water (25 mL). The mixture was heated to reflux for four hours. The cold mixture was treated with aqueous solution of HCl (10%) till total precipitation, the solid was filtered under vacuum and washed with cold water. White solid, 90% yield, m.p.: 210–211 °C. ^1^H NMR (600 MHz, DMSO-*d*_6_) δ 12.97 (s, 1H), 8.72 (d, *J* = 0.9 Hz, 1H), 7.86–7.83 (m, 1H), 7.78 (dt, *J* = 8.5, 1.0 Hz, 1H), 7.75–7.71 (m, 2H), 7.66 (dd, *J* = 8.8, 0.9 Hz, 1H), 7.63 (ddd, *J* = 7.7, 6.3, 2.4 Hz, 1H), 7.33–7.28 (m, 1H), 7.13–7.09 (m, 1H); ^13^C NMR (151 MHz, DMSO-*d*_6_) δ 167.36, 148.77, 138.88, 131.78, 129.83, 129.53, 128.92, 126.32, 126.26, 124.86, 122.07, 121.69, 120.97, 117.38. MS (HR-ESI) for C_14_H_11_N_2_O_2_ [M + H]^+^, calcd: *m*/*z* 239.0815, found: *m*/*z* 239.0824.

*Methyl 2-(2H-Indazol-2-yl)benzoate* (**18**), A 10 mL microwave vial was charged with 2-(2*H*-indazol-2-yl)benzoic acid (1.26 mmol) and 5 mL of solution of HCl in MeOH (15%). The reaction was heated at 120 °C under microwave irradiation for 25 min. Then the reaction was neutralized with 10% solution of NaHCO_3_ and extracted with dichloromethane (3 × 10 mL). The combined organic layers were dried over Na_2_SO_4_ and concentrated under vacuum. The product was purified by column chromatography employing hexanes–dichloromethane–ethyl acetate (50:35:15) as mobile phase. White solid, 36% yield, m.p.: 71–73 °C. The spectroscopic data agree with the previously reported data [48]. ^1^H NMR (600 MHz, CDCl_3_) δ 8.21 (d, *J* = 0.9 Hz, 1H), 7.90 (dd, *J* = 7.7, 1.4 Hz, 1H), 7.76 (dd, *J* = 8.8, 0.9 Hz, 1H), 7.72 (dt, *J* = 8.5, 0.8 Hz, 1H), 7.66–7.59 (m, *J* = 9.2, 7.9, 1.4 Hz, 2H), 7.54 (td, *J* = 7.6, 1.4 Hz, 1H), 7.32 (ddd, *J* = 8.8, 6.6, 1.0 Hz, 1H), 7.12 (ddd, *J* = 8.4, 6.6, 0.6 Hz, 1H), 3.61 (s, 3H); ^13^C NMR (151 MHz, CDCl_3_) δ 166.81, 149.70, 139.80, 132.07, 130.56, 128.82, 128.33, 126.66, 126.33, 123.81, 122.36, 122.34, 120.41, 117.93, 52.46.

### 3.3. Antiprotozoal Activity Assays

*Trichomonas vaginalis* strain GT3, *Giardia intestinalis* isolate IMSS:0981:1 and *Entamoeba histolytica* strain HM1-IMSS were used in all the experiments. Trophozoites of *G. intestinalis* were maintained in a TYI-S-33 medium supplemented with 10% calf serum and bovine bile. *E. histolytica* and *T. vaginalis* trophozoites were maintained in TYI-S-33 medium supplemented with 10% bovine serum. Briefly, 5 × 10^4^ trophozoites of *G. intestinalis* or *T. vaginalis*, or 6 × 10^3^ trophozoites of *E. histolytica* were incubated for 48 h at 37 °C with different concentrations of the compound to be tested, each added as solutions in DMSO. As a negative control, parasites received an equivalent amount of DMSO only, while albendazole (ABZ) and metronidazole (MTZ) were included as positive controls. At the end of the treatment period, the cells were washed and subcultured for another 48 h in fresh medium to which no drug was added. The trophozoites were then counted with a hemocytometer and the 50% inhibitory concentration (IC_50_), together with the respective 95% confidence limit was calculated by Probit analysis. Experiments were carried out in triplicate and repeated at least twice.

### 3.4. Cheminformatic Studies

#### 3.4.1. Molecular Databases

Compounds with IC_50_ annotations against *E. histolytica*, *G. intestinalis* and *T. vaginalis* were retrieved from ChEMBL version 27 [37]. The crude databases were refined by using the following criteria: (a) compounds having structural errors or not readable were eliminated; (b) duplicated compounds were removed to preserve a single structure (the lowest activity value was kept); (c) only compounds with values of IC_50_ < 100 µM were considered; d) counterions were removed from salts and charges were neutralized; (e) inorganic compounds were removed. The curated databases contain 968 compounds against *E. histolytica*, 1211 against *G. intestinalis* and 445 against *T. vaginalis* with pIC_50_ annotations (-log IC_50_[mol/L]). Moreover, 180 benzimidazole derivatives with activity against these same protozoa were taken from previous reports to give 111 derivatives with IC_50_ against *E. histolytica*, 159 compounds against *G. intestinalis* and 134 molecules against *T. vaginalis* [7,23,24,25,26,27,28,29,30,31,32,33,34,35,36]. Compounds were drawing employing ChemDraw Professional 16.0.1.4 software and exported as simplified molecular-input line-entry system (SMILES) with their pIC_50_ annotations. Moreover, indazole derivatives (**1**–**22**) were processed employing the same methodology.

#### 3.4.2. Property Space

A set of six properties were calculated for all compounds under study employing RDKit 2019.09.3 implemented in python 3.7.6: Molecular Weight (MW), Rotatable bonds (RBs), Hydrogen Bond Acceptors (HBAs), Hydrogen Bond Donors (HBDs), Topological Polar Surface Area (TPSA), and Octanol-Water Partition Coefficient (logP). Principal Component Analysis (PCA) were carried out employing the OSIRIS DataWarrior software Version 5.2.1 [41]. Violin plots were calculated employing the Seaborn 0.11.1 library implemented in python 3.7.6.

#### 3.4.3. Structure-Activity Similarity (SAS) Maps

SAS maps were calculated by employing the Activity Landscape Plotter V1 [53]. Molecular ACCess System (MACCS) keys fingerprint (166 bits) and Extended Connectivity Fingerprint (ECFP) with a diameter four were used [54,55]. Each map was generated by plotting molecular similarity in the *X*-axis and the absolute value of the activity differences in the *Y*-axis. The Tanimoto coefficient was used to calculate *N*(*N* − 1)/2 pairwise structural similarities (SS*_i,j_*) for each pair of molecules *i* and *j* included in each scaffold class (benzimidazole or indazole). Moreover, *N*(*N* − 1)/2 pairwise activity differences, determined by the equation |ΔA(T)*_i,j_*| = |A(T)*_i_* − A(T)*_j_*|, were calculated for indazole and benzimidazole datasets; where A(T)*_i_* and A(T)*_j_* are the activities of the *i*th and *j*th molecules (*j* > *i*), in pIC_50_, tested against the target parasite T [42]. SAS maps were divided in four regions employing a threshold of two units on activity difference (100-fold in potency) and the median similarity of the total datapoints plotted in each map (including indazole and benzimidazole derivatives). Briefly, Regions I and II are associated with scaffold hopping (or side chain hopping) and smooth SAR, respectively, whereas Region IV indicates discontinuous SAR (Figure 4).

## 4. Conclusions

A one-pot procedure including a combination of ultrasound synthesis under neat conditions and a Cadogan’s cyclization was implemented for the synthesis of 2-phenyl-2*H*-indazole derivatives. The one-pot method leads to similar or better yields as compared to our previous reports. The biological assays revealed that electron withdrawing groups at the 2-phenyl ring are favorable for antiprotozoal activity. Furthermore, although the SAR information obtained from indazole derivatives as antiprotozoals is still limited, cheminformatic analysis for the 22 compounds studied, highlight their potent values and their smooth SAR nature as compared to reference databases. More studies are needed to expand the activity landscape of indazole derivatives; however, these results represent a valuable start-point toward the optimization of indazole derivatives as antiprotozoals.

## Figures and Tables

**Table 1:** Chemical yield (%) for 2-phenyl-2*H*-indazole derivatives synthesized by ultrasound assisted one-pot procedure, and representative examples of these same compounds synthesized by the conventional method.

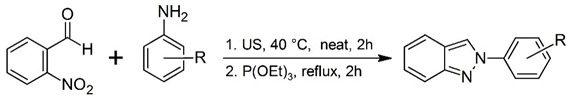			
Compound	R	Ultrasound Assisted One-Pot Procedure	Previously Reported by the Conventional Method [8]
**1**	H	92	60
**2**	4-Cl	62	52
**3**	4-OCH_3_	57	52
**4**	4-COOCH_3_	39	38
**5**	4-F	71	50
**6**	4-CF_3_	43	6
**9**	3-Cl	53	-
**10**	3-OCH_3_	45	-
**11**	3-COOCH_3_	51	-
**12**	3-F	51	-
**13**	3-CF_3_	46	-
**16**	2-Cl	55	-
**17**	2-OCH_3_	69	-
**18a**	2-CN	32 ^1^	-
**19**	2-F	54	-
**20**	2-CF_3_	26	-

^1^ The reaction mixture was ultrasonicated by four cycles of 1 h at 40 °C (*vide infra*).

**Table 2:** Antiprotozoal activity of the 2-pheny-2*H*-indazole derivatives synthesized (IC_50_ [µM] ± SD).

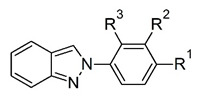						
Compound	R ^1^	R ^2^	R ^3^	*E. histolytica*	*G. intestinalis*	*T. vaginalis*
**1** ^1^	H	H	H	0.080 ± 0.004	0.113 ± 0.022	0.118 ± 0.022
**2** ^1^	Cl	H	H	0.042 ± 0.003	0.063 ± 0.003	0.107 ± 0.003
**3** ^1^	OCH_3_	H	H	0.154 ± 0.016	0.205 ± 0.006	0.372 ± 0.016
**4** ^1^	COOCH_3_	H	H	0.022 ± 0.003	0.063 ± 0.006	0.107 ± 0.006
**5**	F	H	H	0.075 ± 0.007	0.071 ± 0.007	0.118 ± 0.020
**6**	CF_3_	H	H	0.053 ± 0.000	0.034 ± 0.005	0.084 ± 0.016
**7** ^1^	OH	H	H	0.074 ± 0.010	0.119 ± 0.007	0.157 ± 0.014
**8** ^1^	COOH	H	H	0.097 ± 0.006	0.193 ± 0.012	0.327 ± 0.018
**9**	H	Cl	H	0.070 ± 0.012	0.083 ± 0.012	0.101 ± 0.006
**10**	H	OCH_3_	H	0.078 ± 0.010	0.056 ± 0.010	0.120 ± 0.019
**11**	H	COOCH_3_	H	0.036 ± 0.000	0.054 ± 0.003	0.069 ± 0.008
**12**	H	F	H	0.097 ± 0.010	0.087 ± 0.003	0.092 ± 0.010
**13**	H	CF_3_	H	0.061 ± 0.005	0.050 ± 0.005	0.069 ± 0.005
**14**	H	OH	H	0.102 ± 0.003	0.105 ± 0.000	0.162 ± 0.000
**15**	H	COOH	H	0.074 ± 0.009	0.053 ± 0.009	0.065 ± 0.009
**16**	H	H	Cl	0.088 ± 0.006	0.031 ± 0.006	0.066 ± 0.012
**17**	H	H	OCH_3_	0.096 ± 0.003	0.083 ± 0.003	0.183 ± 0.006
**18**	H	H	COOCH_3_	0.050 ± 0.003	0.032 ± 0.006	0.109 ± 0.008
**19**	H	H	F	0.092 ± 0.010	0.071 ± 0.007	0.108 ± 0.007
**20**	H	H	CF_3_	0.031 ± 0.011	0.027 ± 0.011	0.128 ± 0.014
**21**	H	H	OH	0.145 ± 0.010	0.074 ± 0.003	0.112 ± 0.010
**22**	H	H	COOH	0.074 ± 0.003	0.023 ± 0.009	0.067 ± 0.006
MTZ ^2^	-	-	-	0.380 ± 0.146	1.226 ± 0.125	0.236 ± 0.016
ABZ ^3^	-	-	-	56.533 ± 18.845	0.037 ± 0.003	1.591 ± 0.011

^1^ IC_50_ values were taken from Pérez-Villanueva et al. 2017 [8]; ^2^ Metronidazole; ^3^ Albendazole.

## Data Availability

The data presented in this study are available on request from the corresponding author.

## Supplementary Materials

The following are available online, Figures S1–S23 ^1^H NMR and ^13^C NMR for 2-phenyl-2*H*-indazole derivatives synthesized (**1**–**22** and **18a**).

## Sample Availability

Samples of the compounds **1**–**22** and **18a** are available from the authors.
