# Supplementary material for: Synthesis, Antiprotozoal Activity, and Cheminformatic Analysis of 2-Phenyl-2H-Indazole Derivatives
Source: Molecules. 2021 Apr 8;26(8):2145. doi: 10.3390/molecules26082145 (PMC8068258; doi:10.3390/molecules26082145)
Supplement: Supplementary file 1 [file molecules-26-02145-s001.pdf]

Supporting Information

Synthesis, antiprotozoal activity, and cheminformatic analysis of 2-phenyl-2*H*-indazole derivatives

Karen Rodríguez-Villar [1], Lilián Yépez-Mulia [2], Miguel Cortés-Gines [3], Jacobo David Aguilera-Perdomo [3], Edgar A. Quintana-Salazar [3], Kevin Samael Olascoaga Del Angel [4], Francisco Cortés-Benítez [3], Juan Francisco Palacios-Espinosa [3], Olivia Soria-Arteche [3] and Jaime Pérez-Villanueva [3,*]

[1] Doctorado en Ciencias Biológicas y de la Salud, Universidad Autónoma Metropolitana (UAM), Ciudad de México 04960, Mexico; qkarenrodv@hotmail.com

[2] Unidad de Investigación Médica en Enfermedades Infecciosas y Parasitarias, UMAE Hospital de Pediatría, Centro Médico Siglo XXI, Instituto Mexicano del Seguro Social, Ciudad de México 06720, Mexico; lilianyepez@yahoo.com

[3] Departamento de Sistemas Biológicos, División de Ciencias Biológicas y de la Salud, Universidad Autónoma Metropolitana-Xochimilco (UAM-X), Ciudad de México 04960, Mexico; mcortes@pharmometrica.com.mx (M.C.-G.); jacoboaguilera.96@gmail.com (J.D.A.-P); edgarqsl2811@gmail.com (E.A.Q.-S); jcortesb@correo.xoc.uam.mx (F.C.-B); jpalacios@correo.xoc.uam.mx (J.F.P.-E.); soriao@correo.xoc.uam.mx (O.S.-A.);

[4] Doctorado en Biología experimental, Universidad Autónoma Metropolitana (UAM), Ciudad de México 04960, Mexico; olaskuaga@gmail.com

* Correspondence: jpvillanueva@correo.xoc.uam.mx; Tel.: +525 54 83 72 59; fax: +525 55 94 79 29.

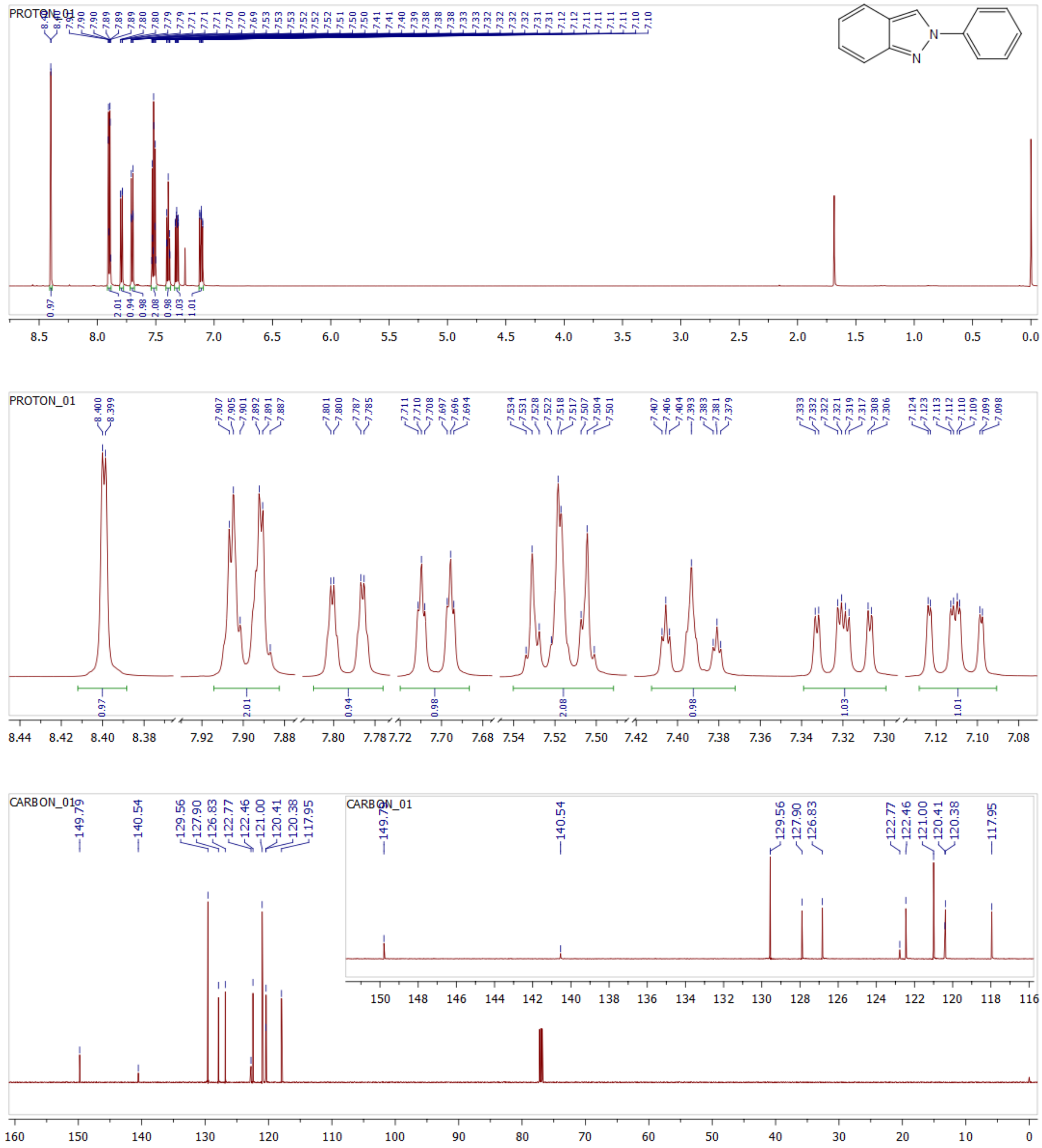

Figure S1. ^{1}H NMR (600 MHz, $CDCl_3$) and ^{13}C NMR (151 MHz, $CDCl_3$) for 2-phenyl-2*H*-indazole (**1**).

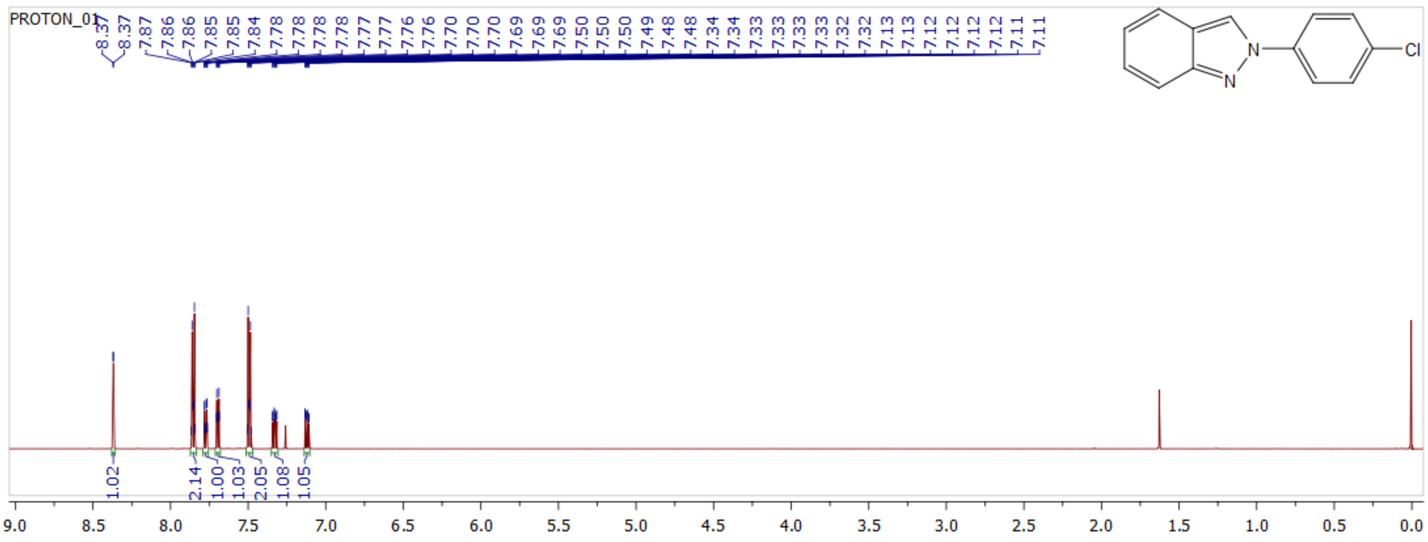

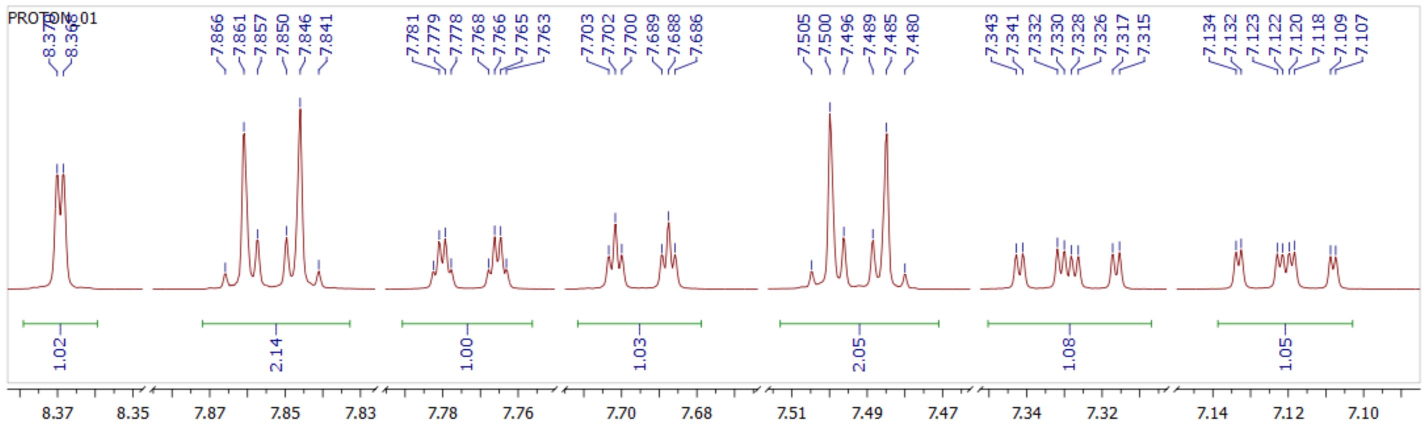

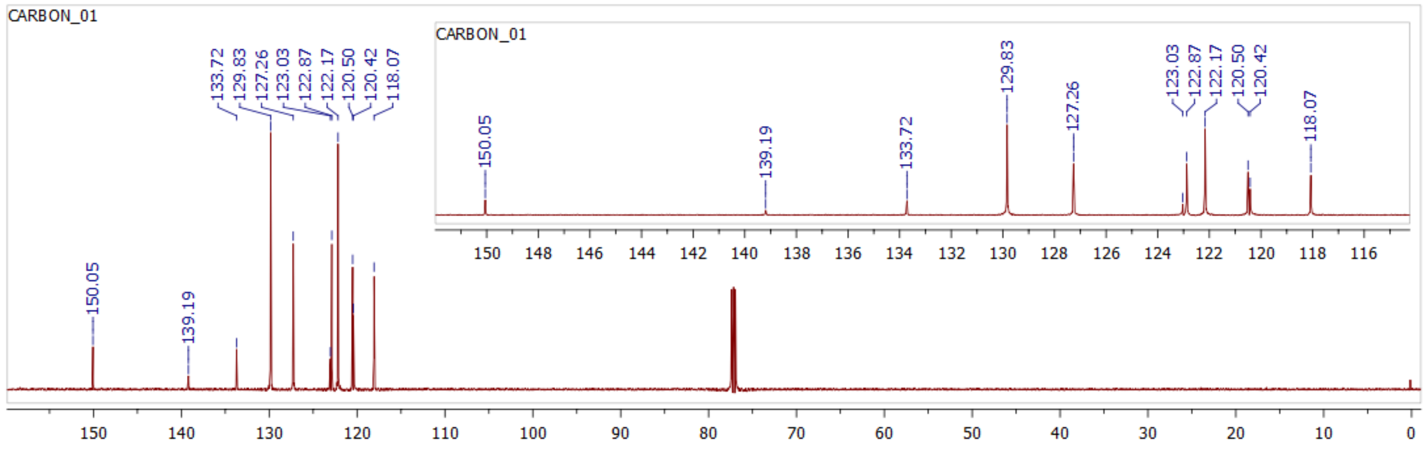

Figure S2. ^{1}H NMR (600 MHz, $CDCl_3$) and ^{13}C NMR (151 MHz, $CDCl_3$) for 2-(4-chlorophenyl)-2*H*-indazole (**2**)

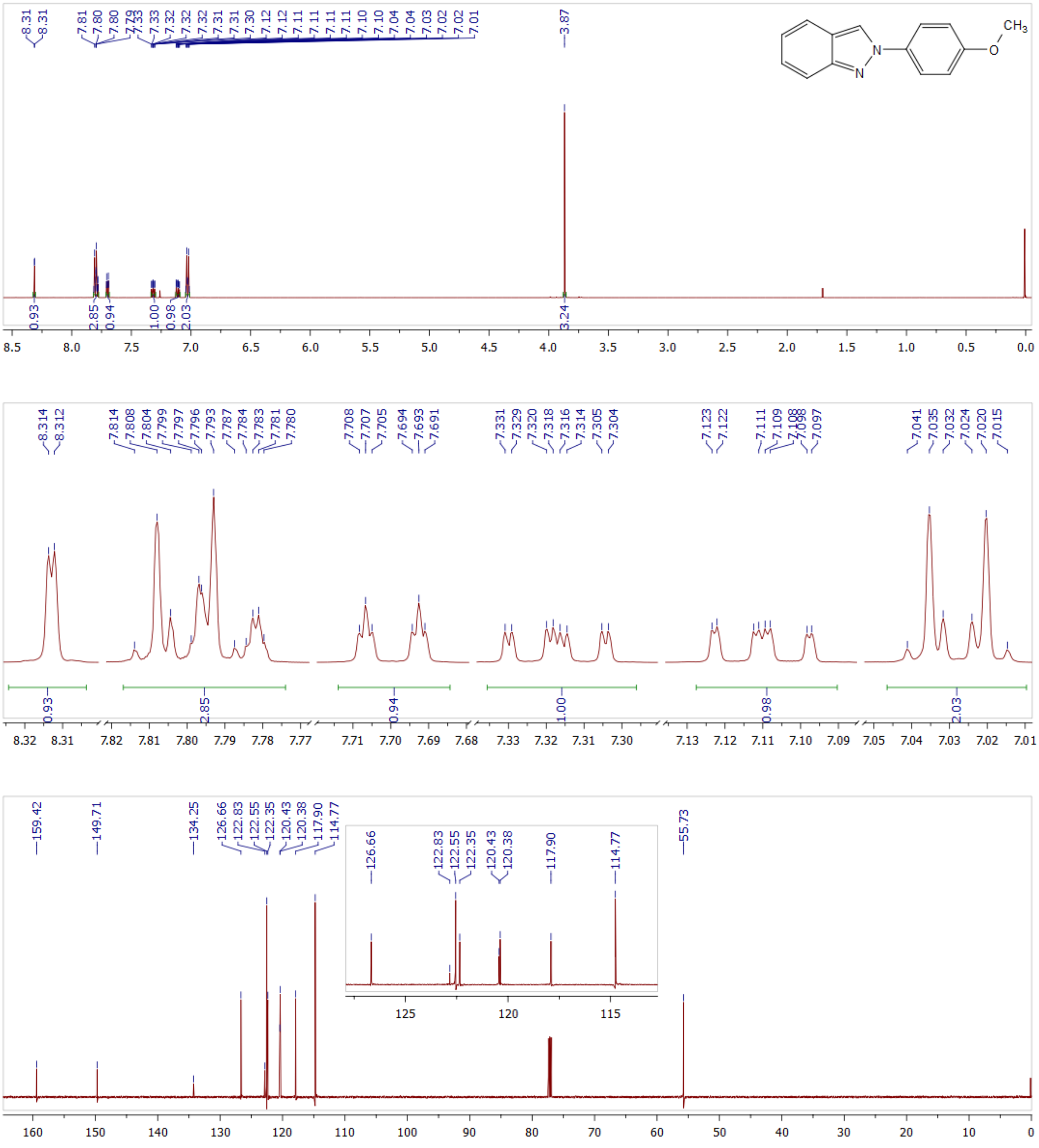

Figure S3. ^{1}H NMR (600 MHz, $CDCl_3$) and ^{13}C NMR (151 MHz, $CDCl_3$) for 2-(4-methoxyphenyl)-2*H*-indazole (**3**).

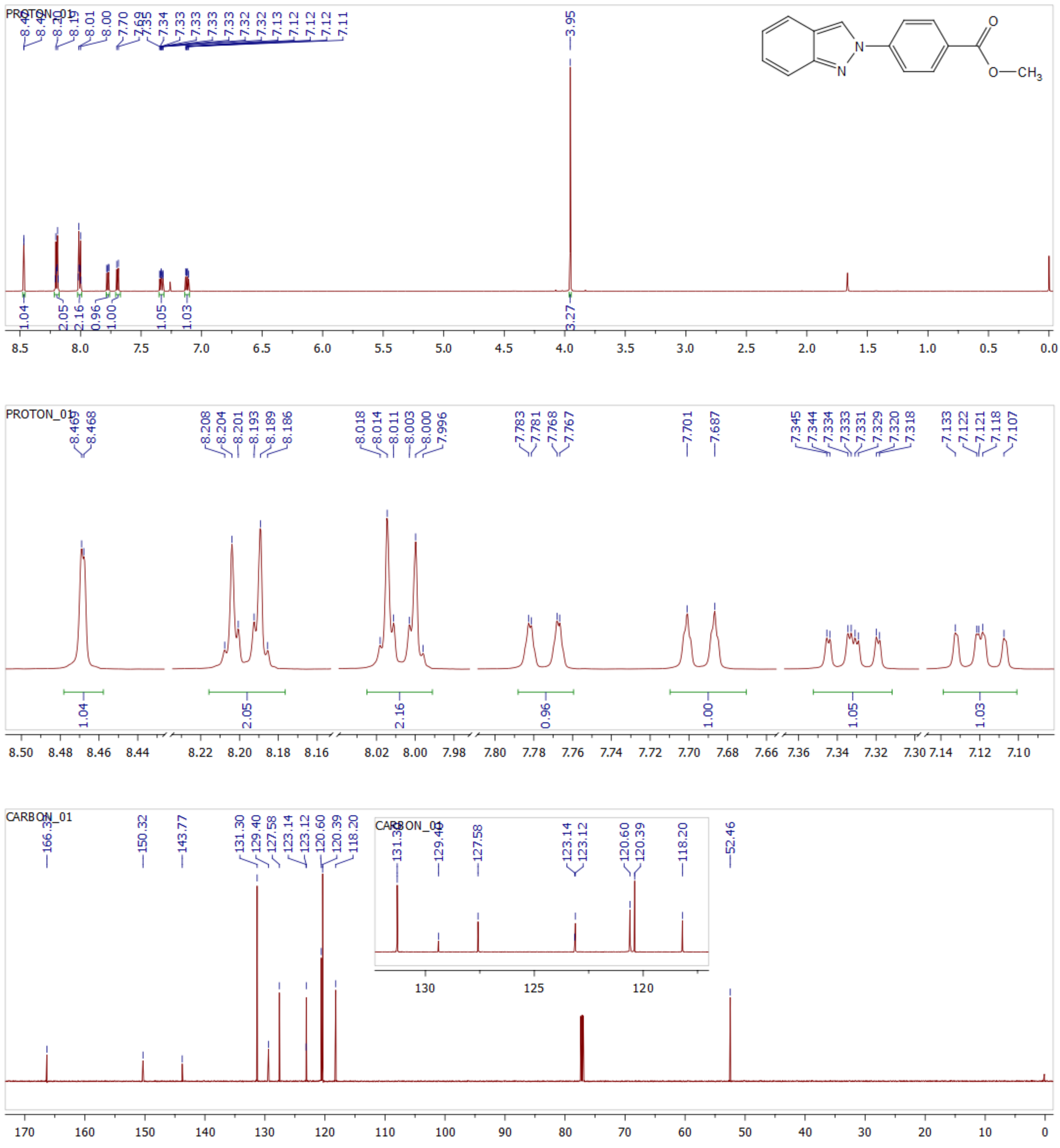

Figure S4. ^{1}H NMR (600 MHz, $CDCl_3$) and ^{13}C NMR (151 MHz, $CDCl_3$) for methyl 4-(2*H*-indazol-2-yl)benzoate (**4**).

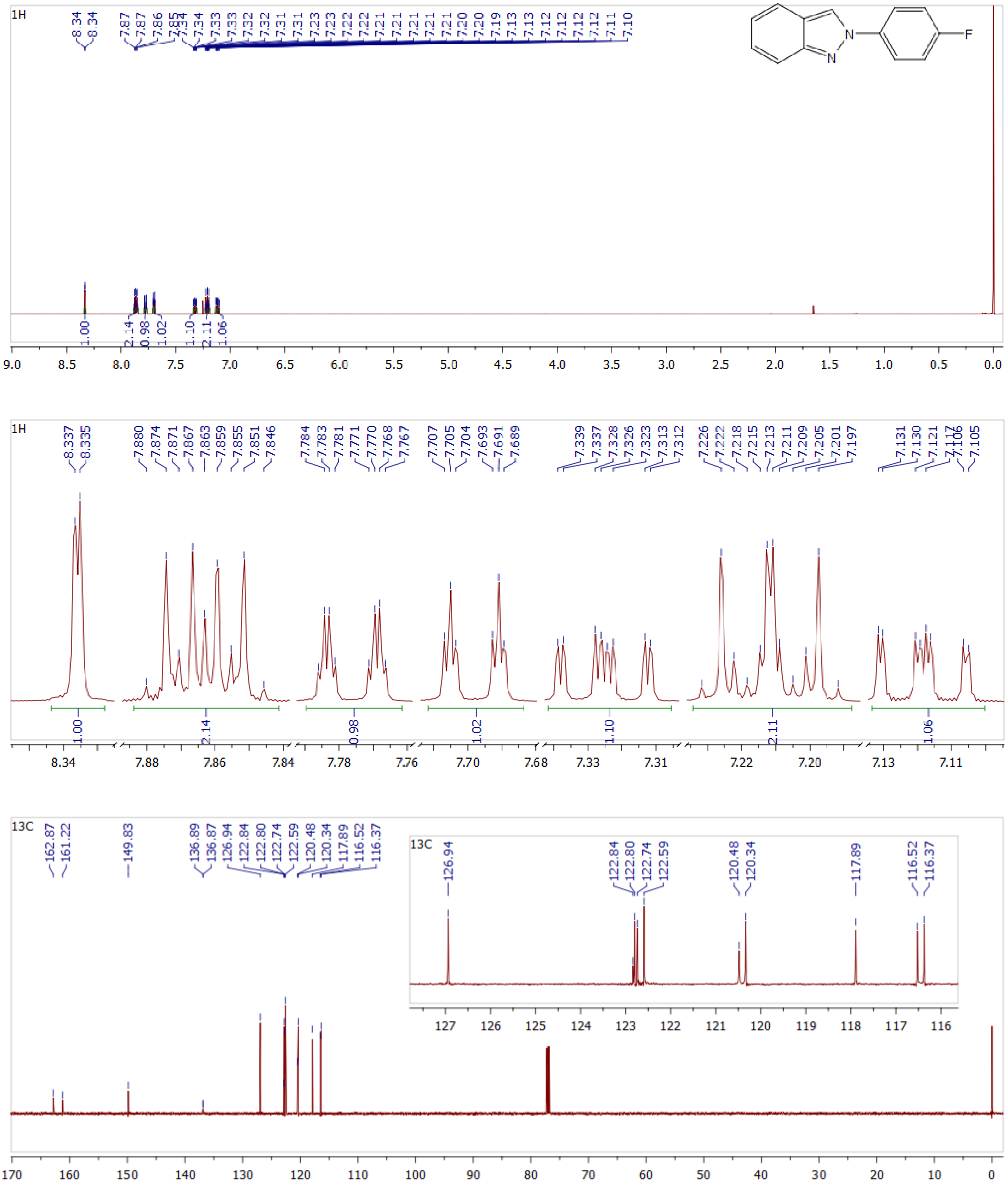

Figure S5. ^{1}H NMR (600 MHz, $CDCl_3$) and ^{13}C NMR (151 MHz, $CDCl_3$) for 2-(4-fluorophenyl)-2*H*-indazole (**5**).

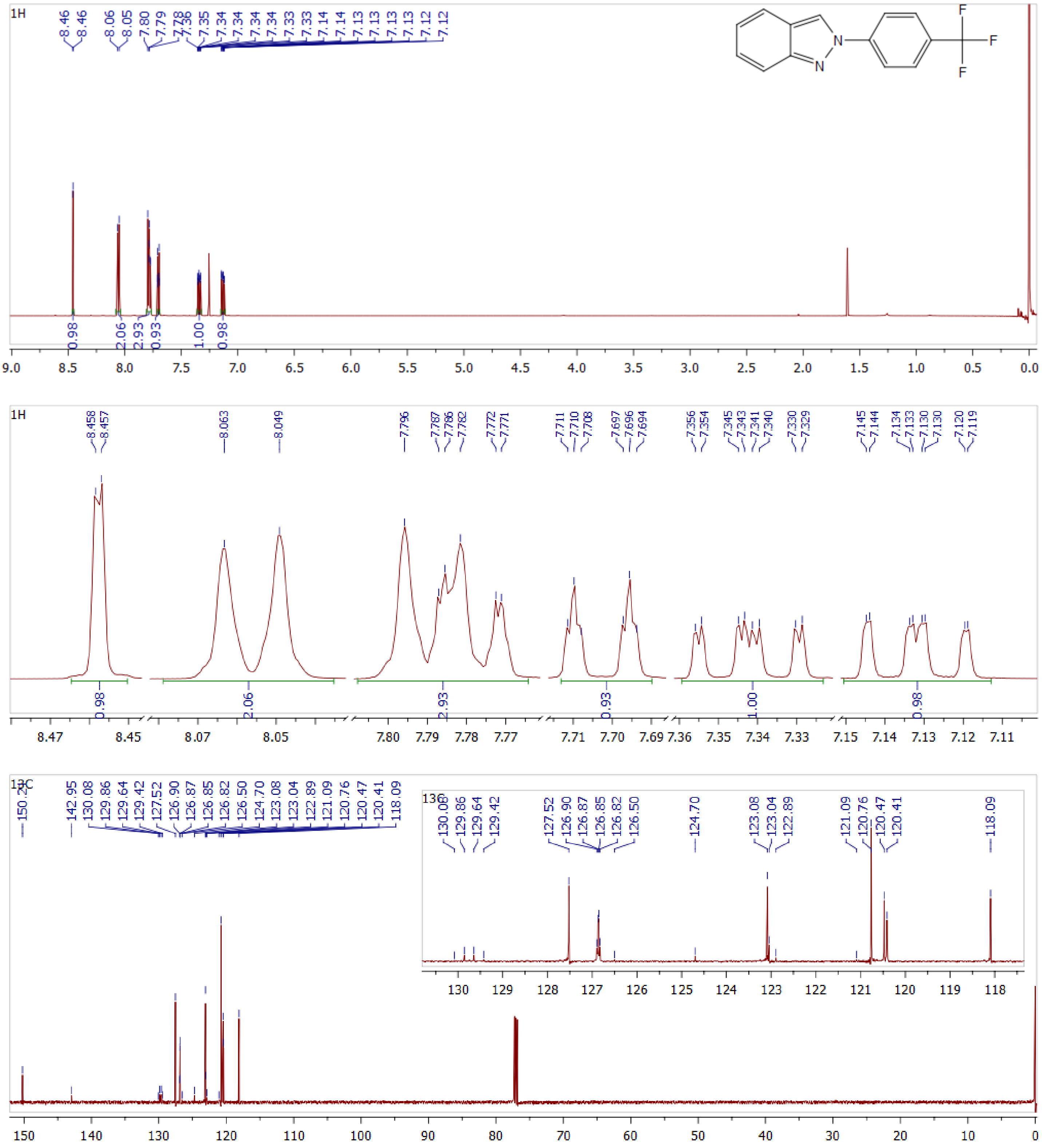

Figure S6. ^{1}H NMR (600 MHz, $CDCl_3$) and ^{13}C NMR (151 MHz, $CDCl_3$) for 2-[4-(trifluoromethyl)phenyl]-2*H*-indazole (**6**).

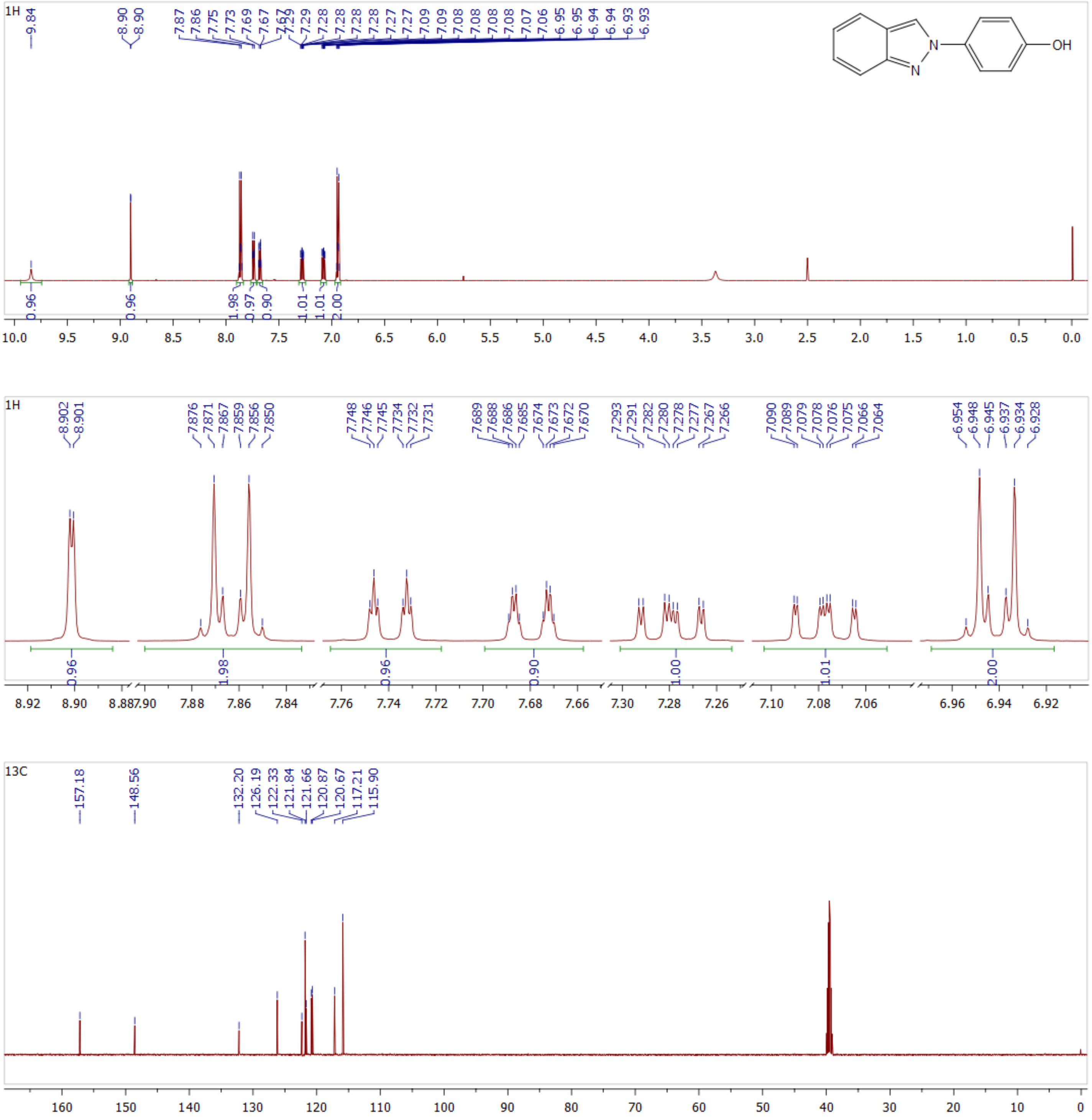

Figure S7. ^{1}H NMR (600 MHz, DMSO-d_6) and ^{13}C NMR (151 MHz, DMSO-d_6) for 4-(2*H*-indazol-2-yl) phenol (**7**).

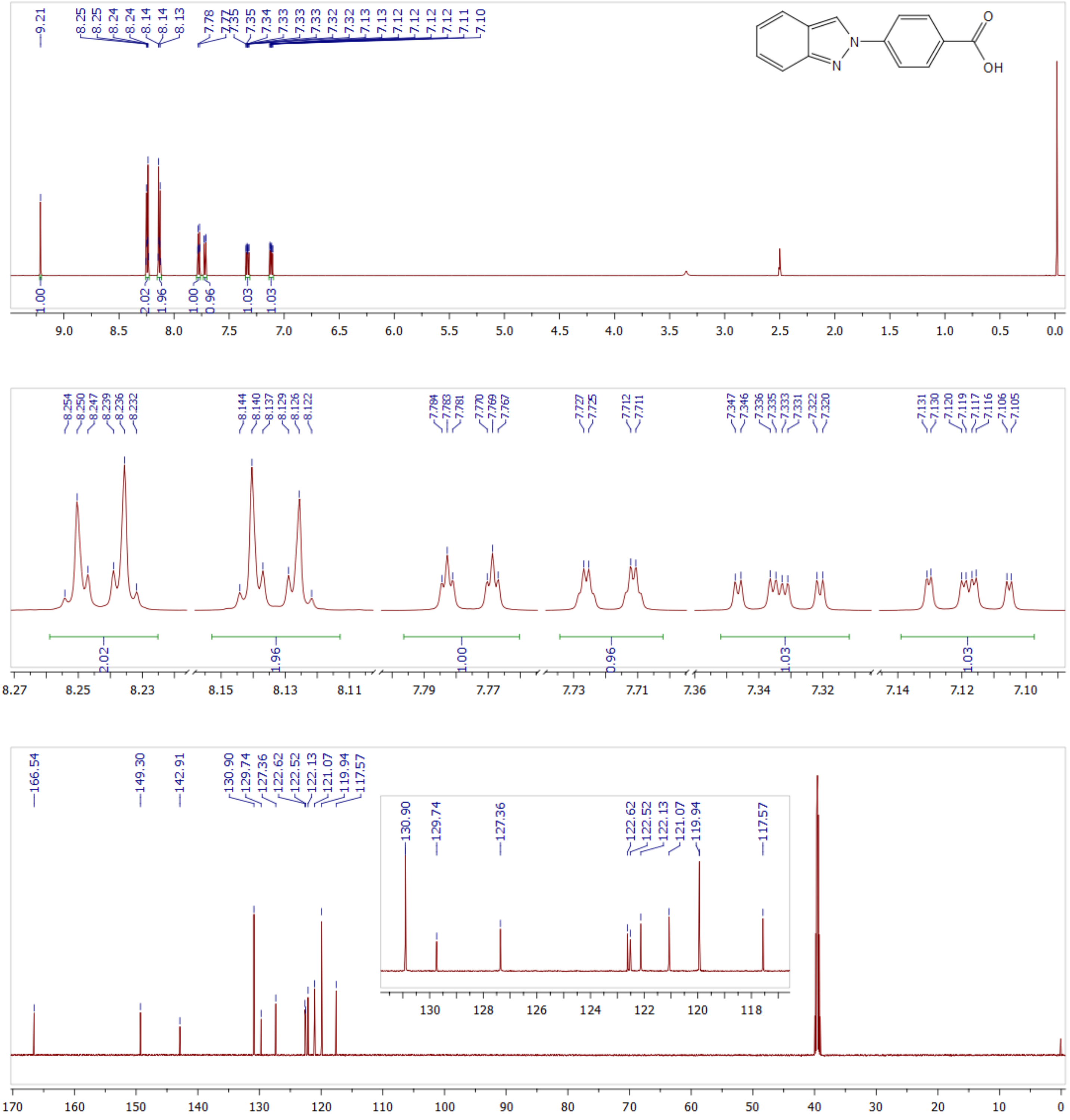

Figure S8. ^{1}H NMR (600 MHz, DMSO-d_6) and ^{13}C NMR (151 MHz, DMSO-d_6) for 4-(2*H*-indazol-2-yl) benzoic acid (**8**).

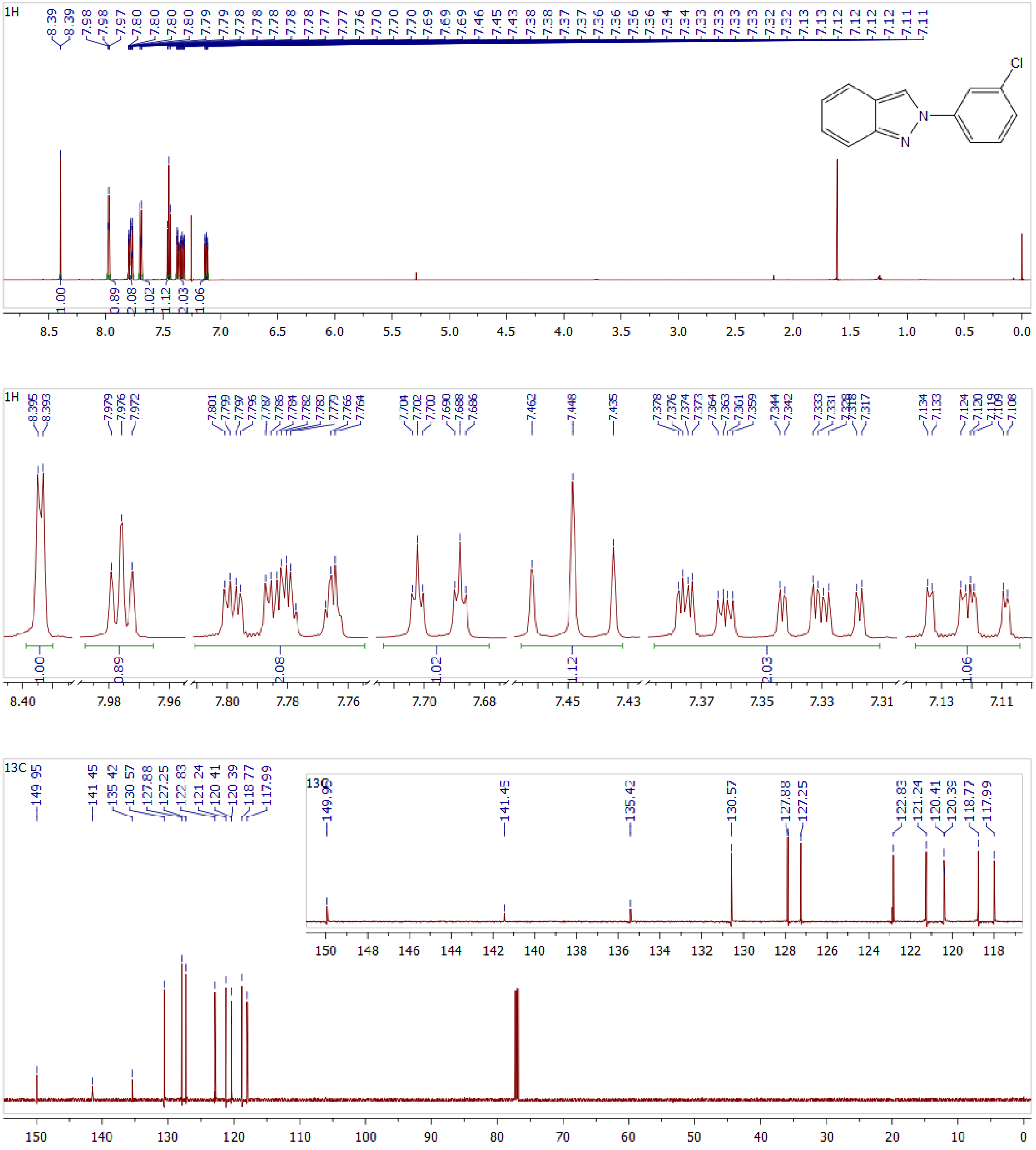

Figure S9. ^{1}H NMR (600 MHz, $CDCl_3$) and ^{13}C NMR (151 MHz, $CDCl_3$) for 2-(3-chlorophenyl)-2*H*-indazole (**9**).

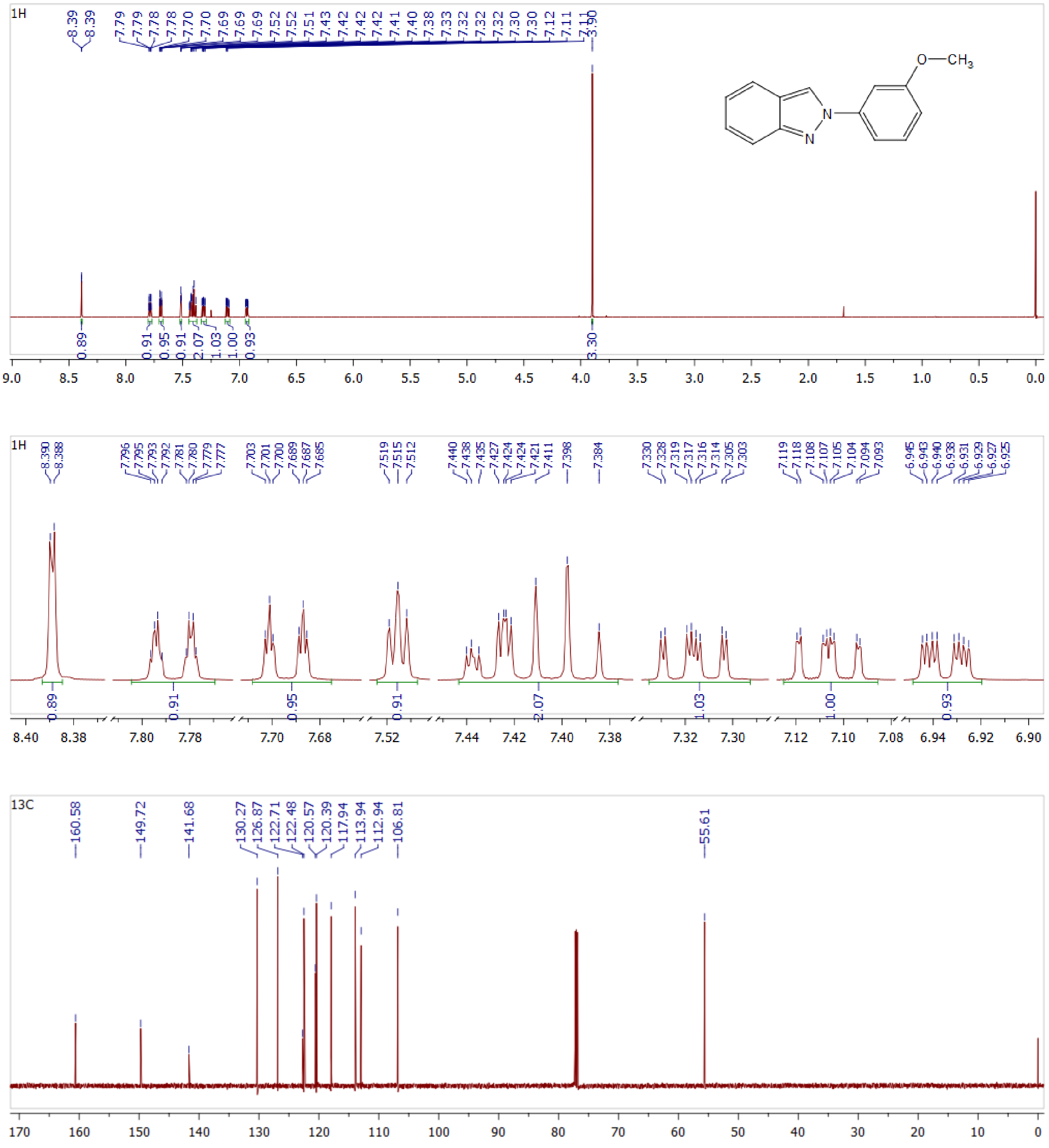

Figure S10. ^{1}H NMR (600 MHz, $CDCl_3$) and ^{13}C NMR (151 MHz, $CDCl_3$) for 2-(3-methoxyphenyl)-2*H*-indazole (**10**).

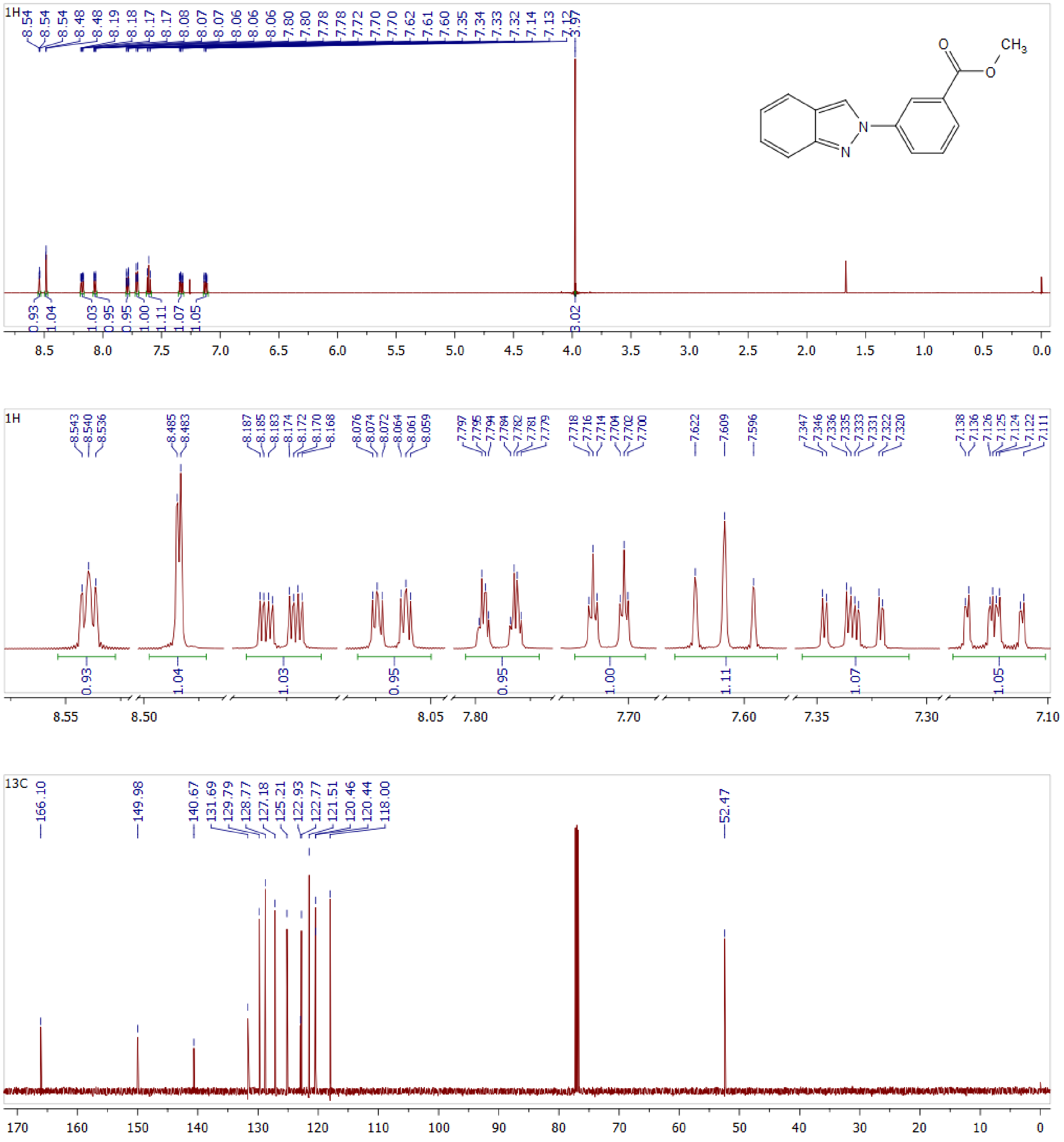

Figure S11. ^{1}H NMR (600 MHz, $CDCl_3$) and ^{13}C NMR (151 MHz, $CDCl_3$) for methyl 3-(2*H*-indazol-2-yl)benzoate (**11**).

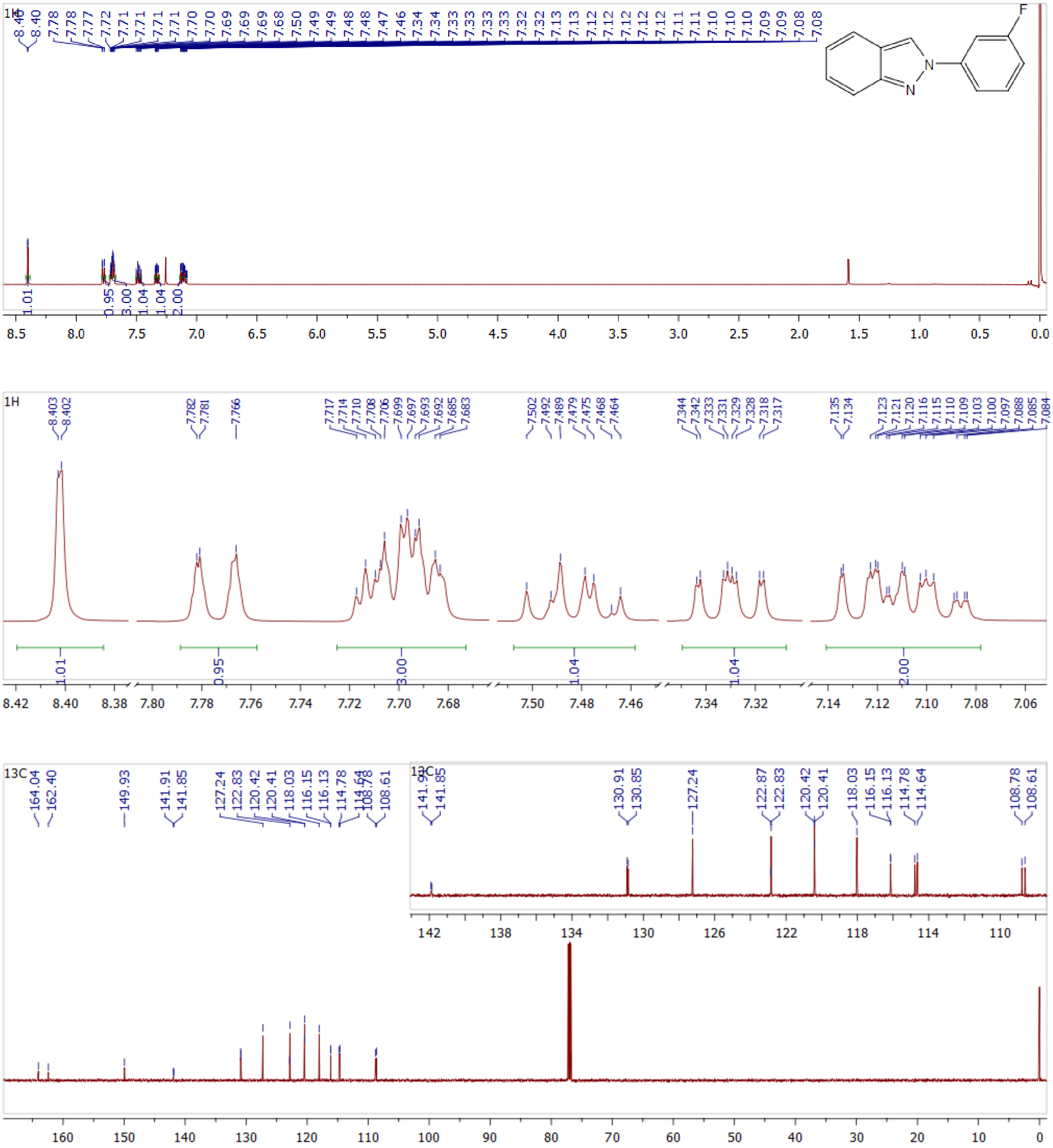

Figure S12. ^{1}H NMR (600 MHz, $CDCl_3$) and ^{13}C NMR (151 MHz, $CDCl_3$) for 2-(3-fluorophenyl)-2*H*-indazole (**12**).

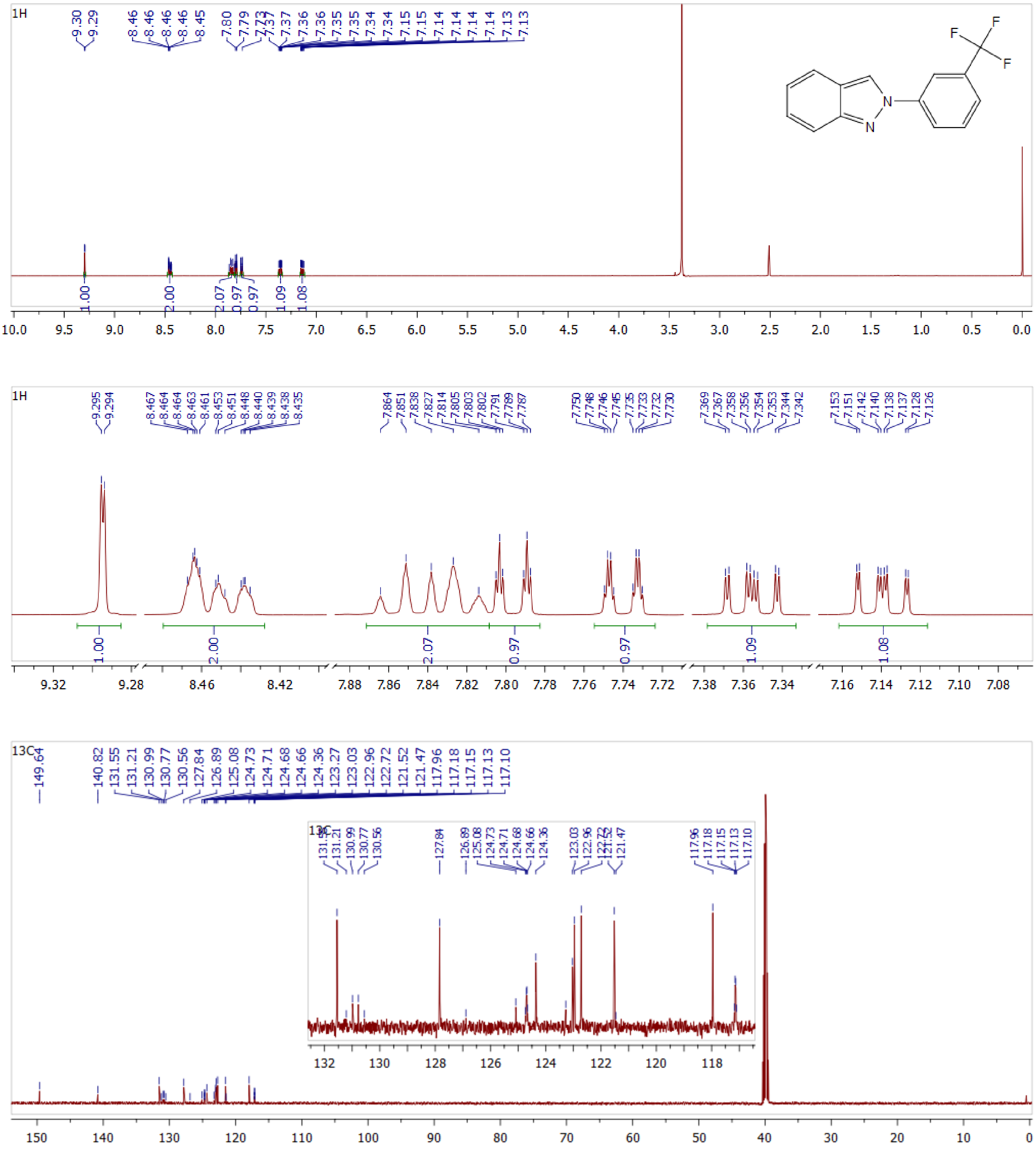

Figure S13. ^{1}H NMR (600 MHz, DMSO-d_6) and ^{13}C NMR (151 MHz, DMSO-d_6) for 2-[3-(trifluoromethyl)phenyl]-2*H*-indazole (**13**).

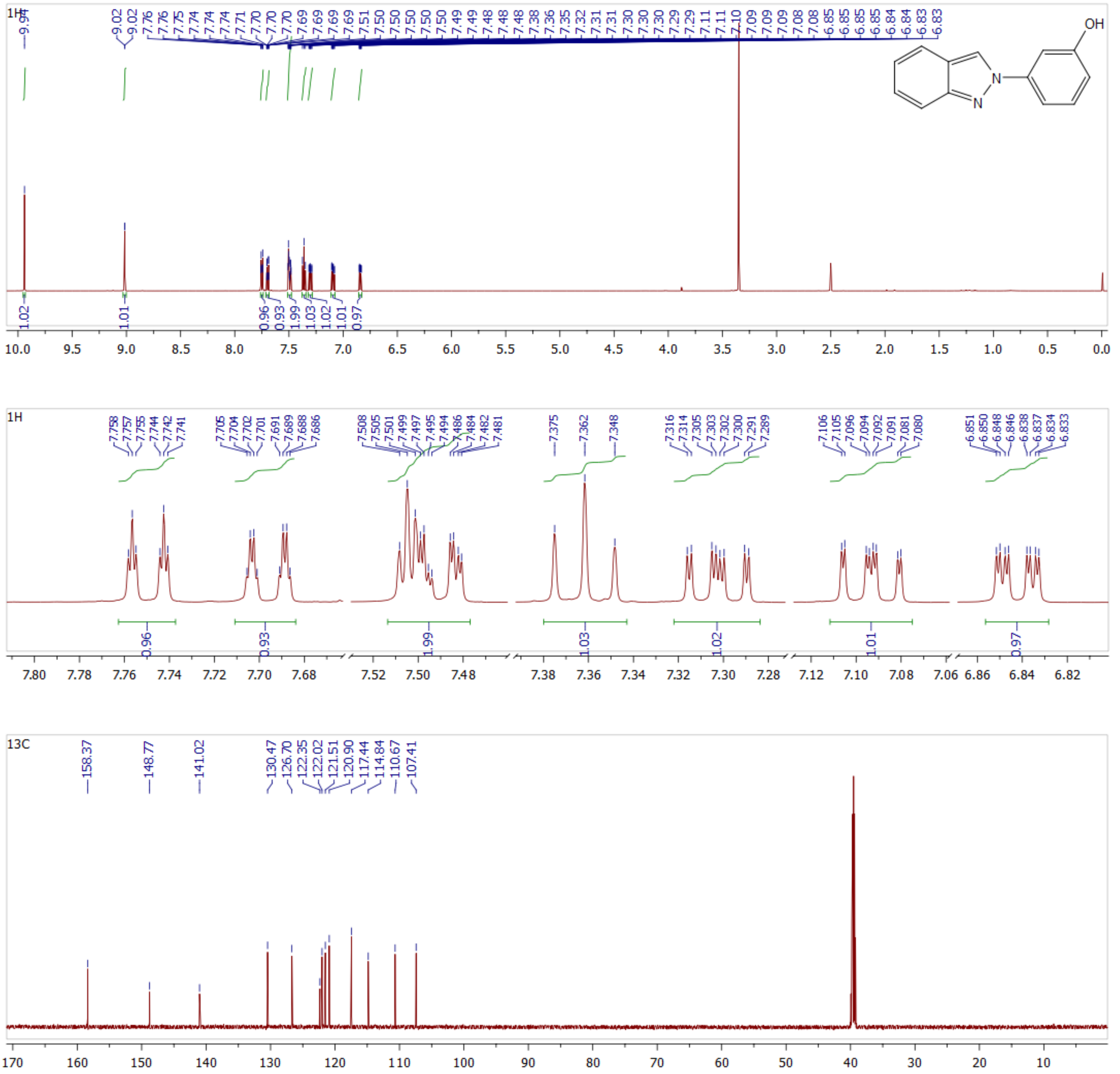

Figure S14. ^{1}H NMR (600 MHz, DMSO-d_6) and ^{13}C NMR (151 MHz, DMSO-d_6) for 3-(2*H*-indazol-2-yl)phenol (**14**).

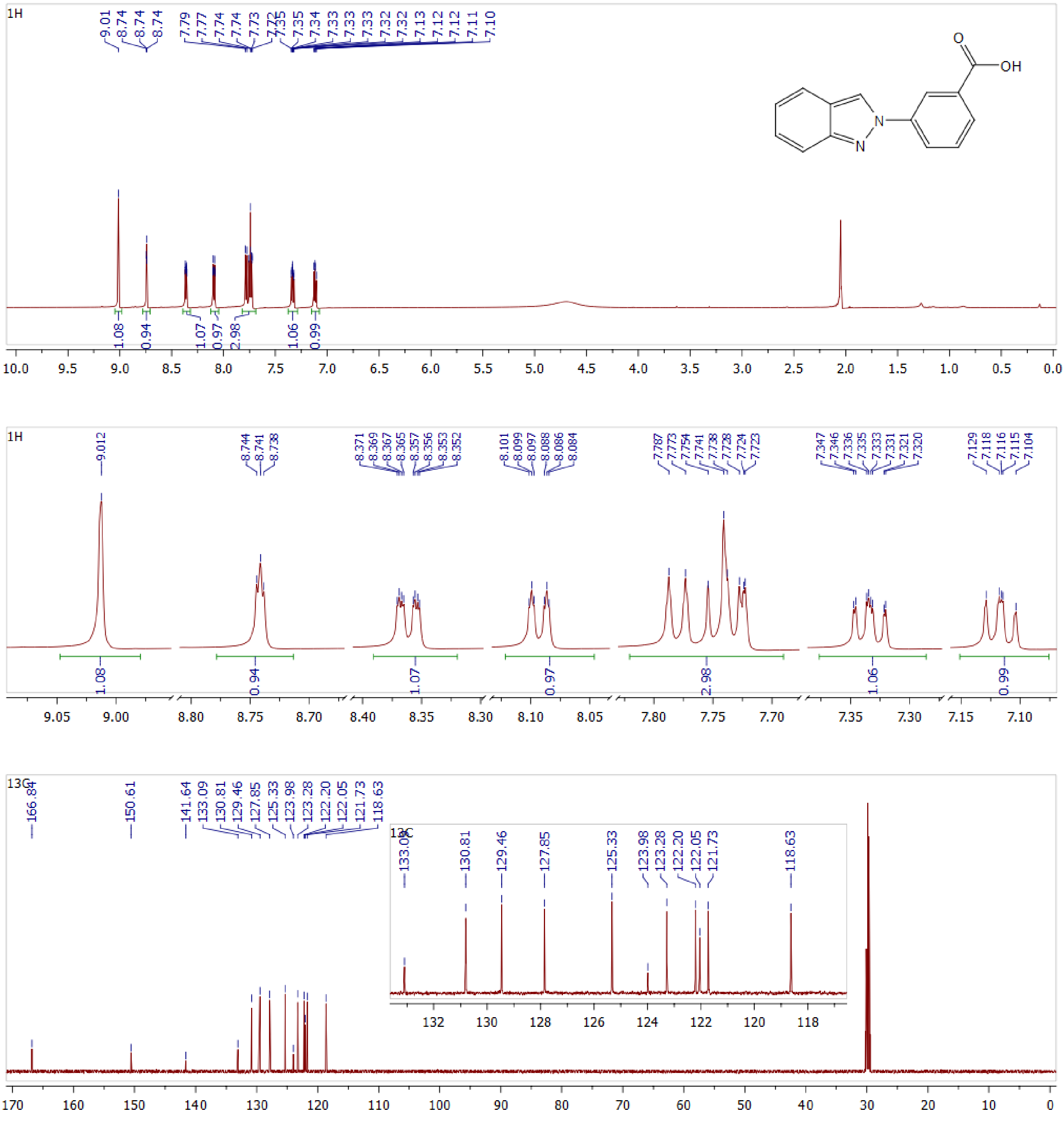

Figure S15. ^{1}H NMR (600 MHz, acetone-d_6) and ^{13}C NMR (151 MHz, acetone-d_6) for 3-(2*H*-indazol-2-yl)benzoic acid (**15**).

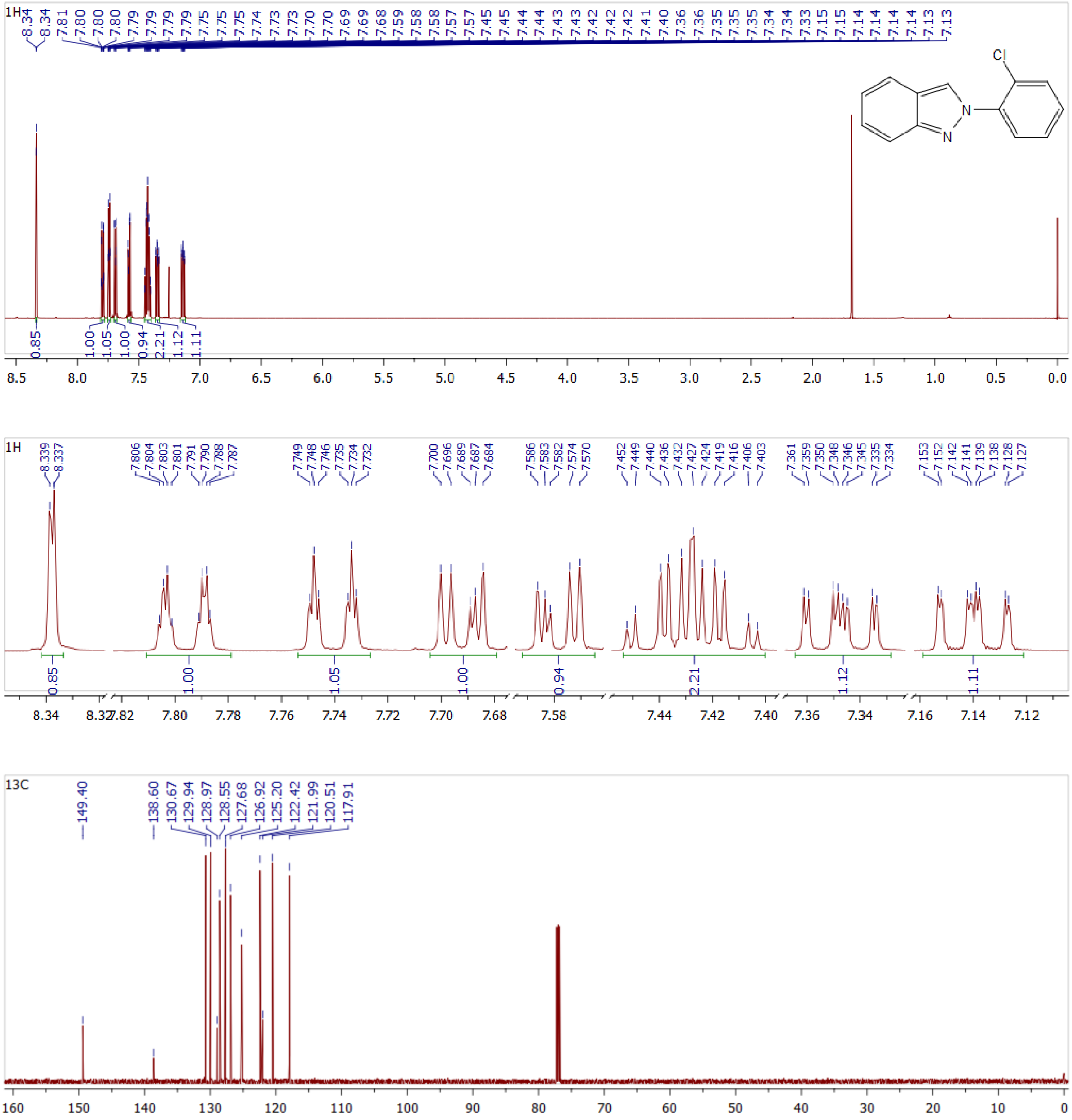

Figure S16. ^{1}H NMR (600 MHz, $CDCl_3$) and ^{13}C NMR (151 MHz, $CDCl_3$) for 2-(2-chlorophenyl)-2*H*-indazole (**16**).

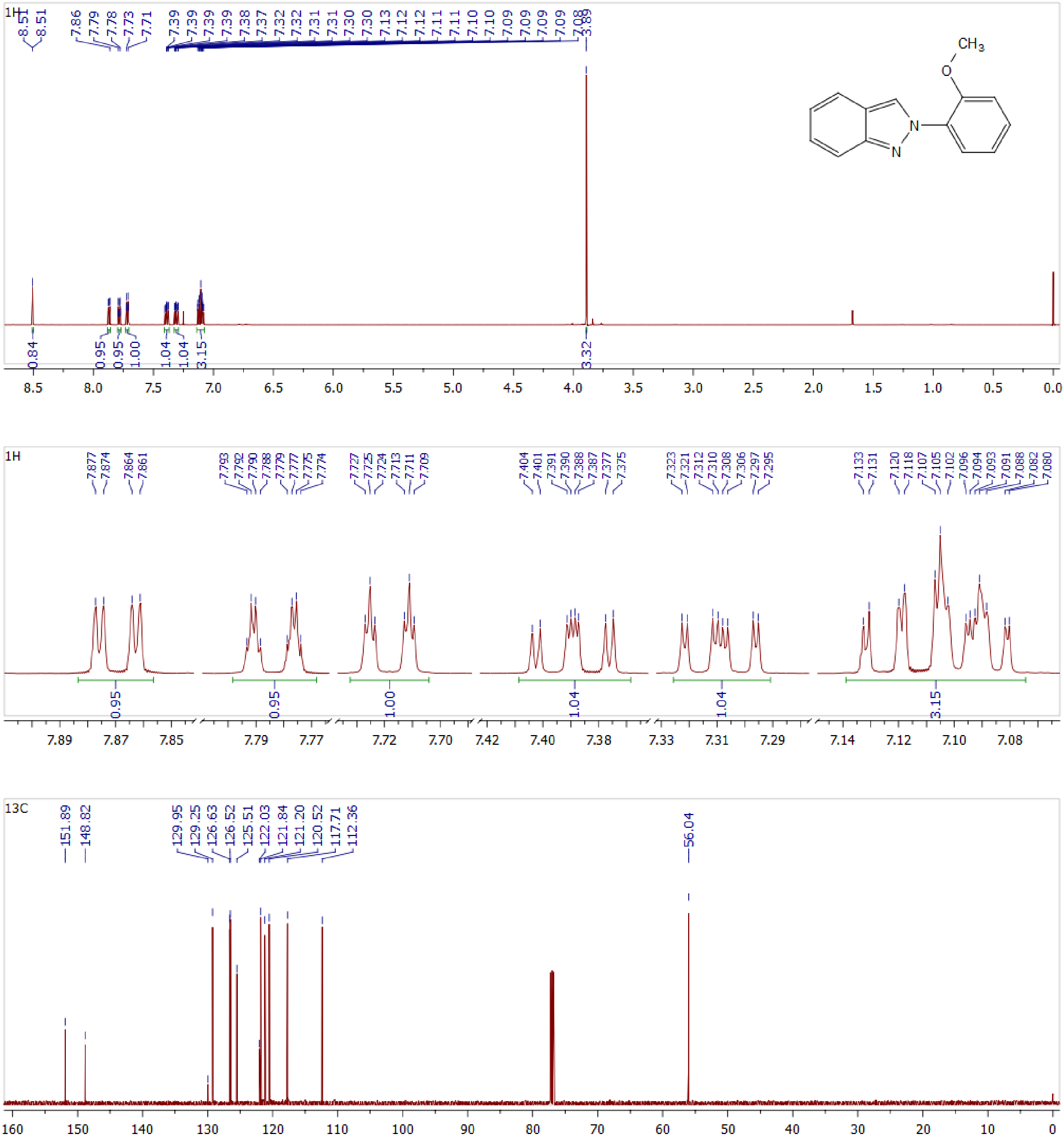

Figure S17. ^{1}H NMR (600 MHz, $CDCl_3$) and ^{13}C NMR (151 MHz, $CDCl_3$) for 2-(2-methoxyphenyl)-2*H*-indazole (**17**).

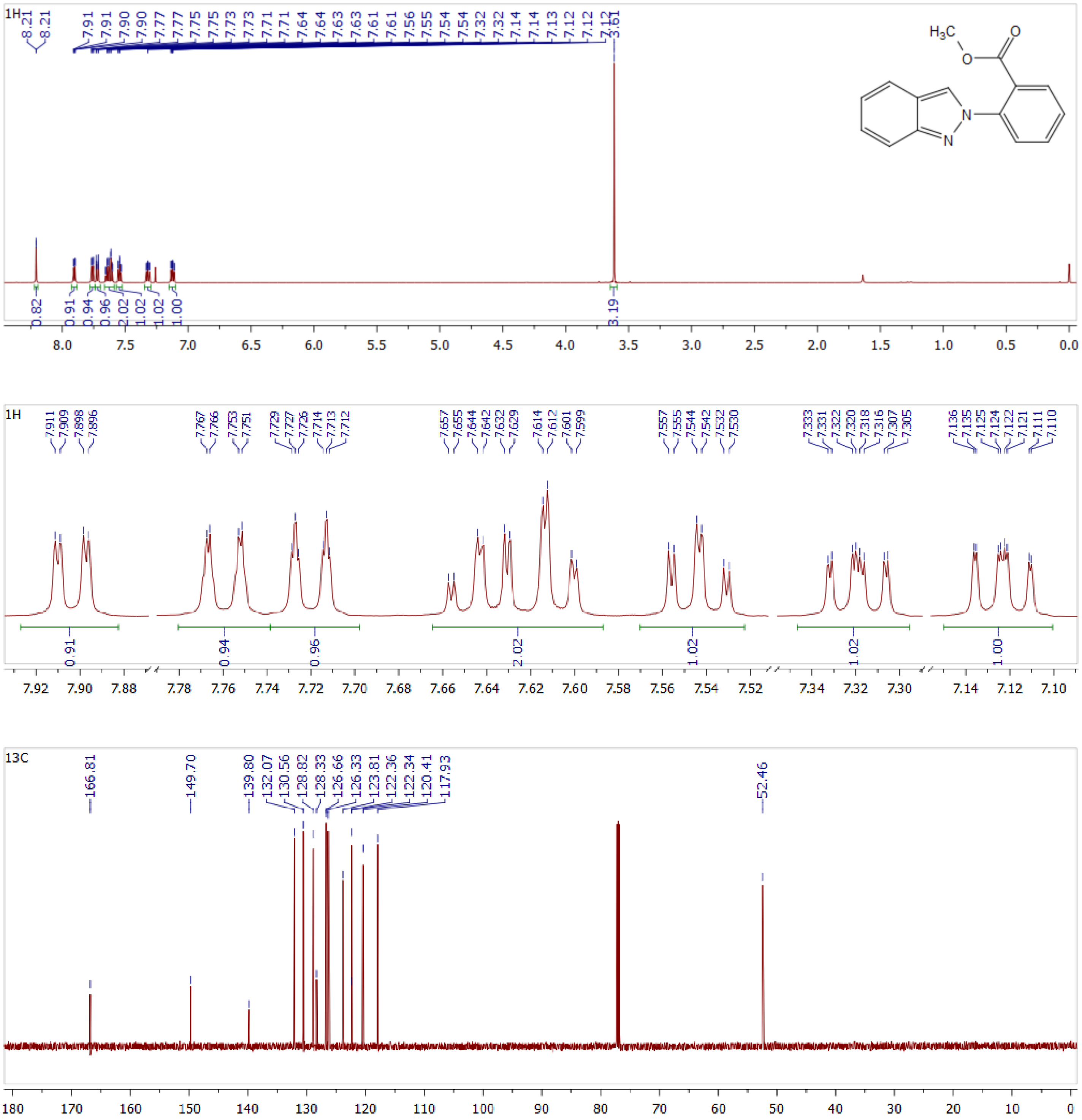

Figure S18. ^{1}H NMR (600 MHz, $CDCl_3$) and ^{13}C NMR (151 MHz, $CDCl_3$) for methyl 2-(2*H*-indazol-2-yl)benzoate (**18**).

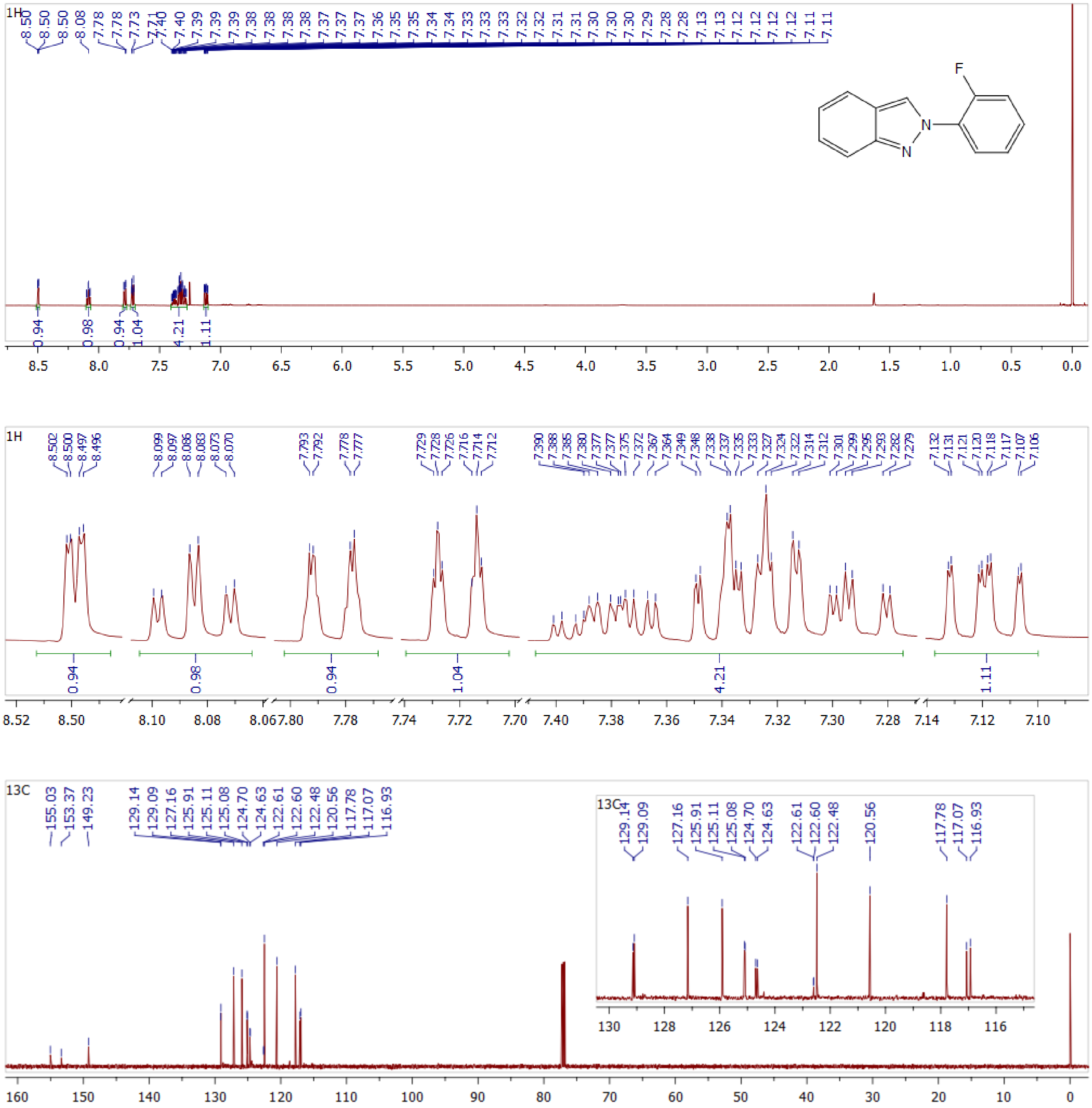

Figure S19. ^{1}H NMR (600 MHz, $CDCl_3$) and ^{13}C NMR (151 MHz, $CDCl_3$) for 2-(2-fluorophenyl)-2*H*-indazole (**19**).

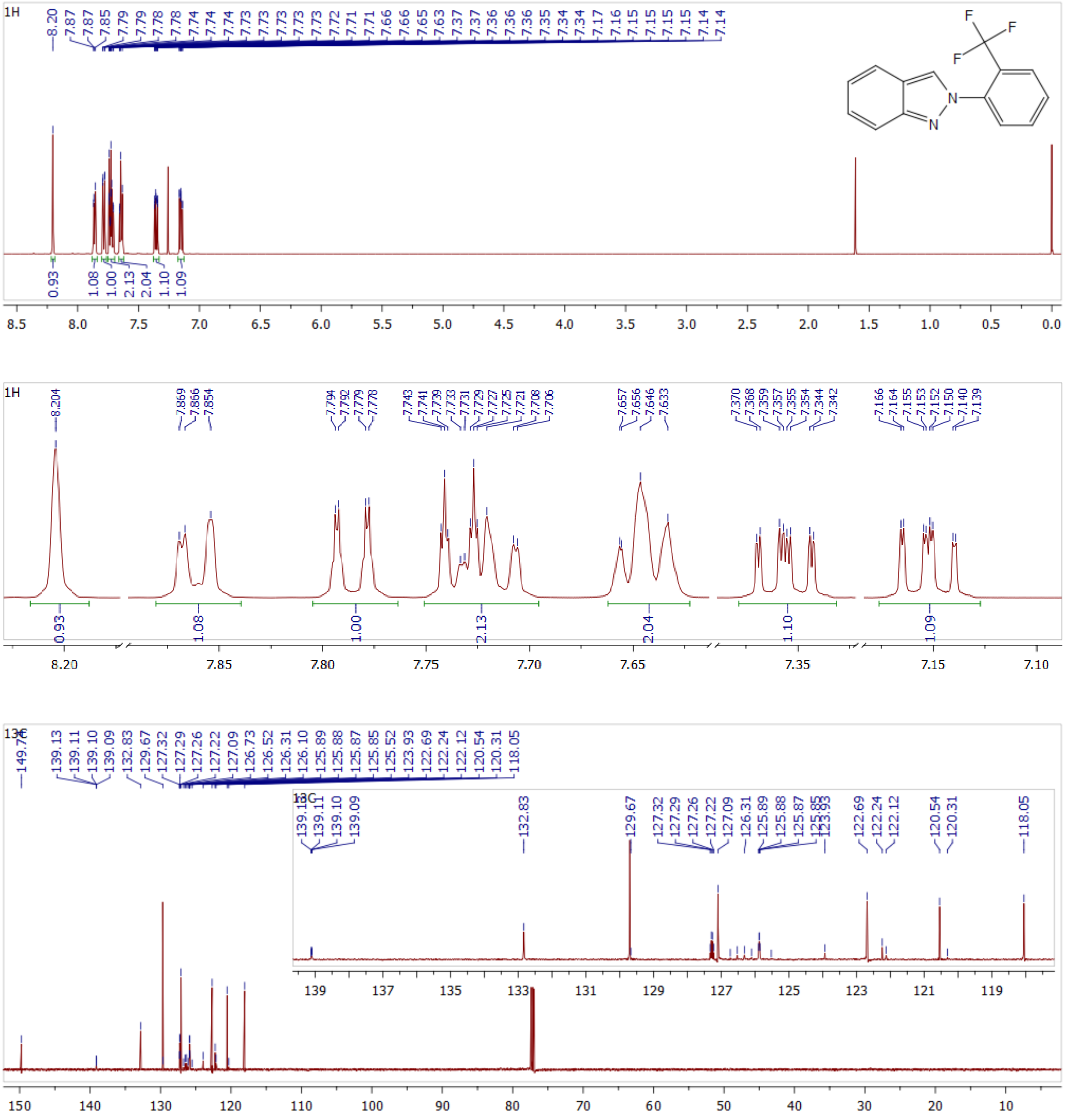

Figure S20. ^{1}H NMR (600 MHz, $CDCl_3$) and ^{13}C NMR (151 MHz, $CDCl_3$) for 2-[2-(trifluoromethyl)phenyl]-2*H*-indazole (**20**).

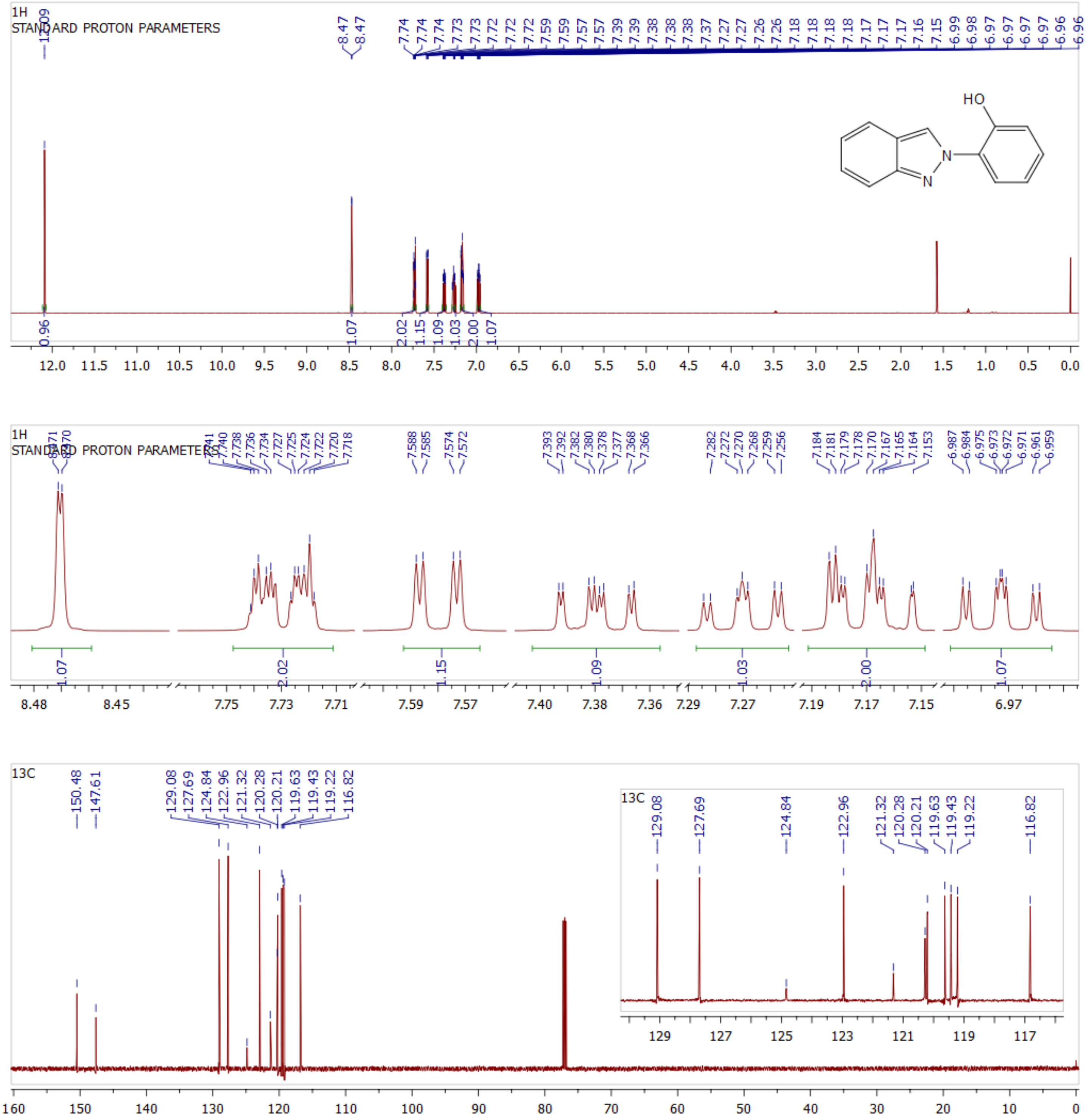

Figure S21. ^{1}H NMR (600 MHz, $CDCl_3$) and ^{13}C NMR (151 MHz, $CDCl_3$) for 2-(2*H*-indazol-2-yl)phenol (**21**).

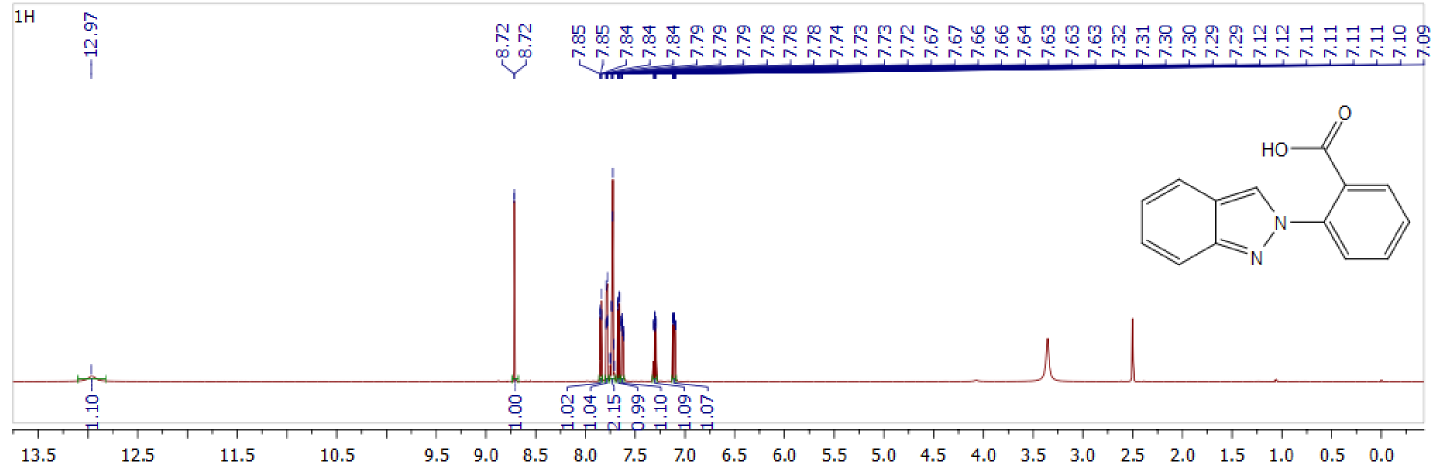

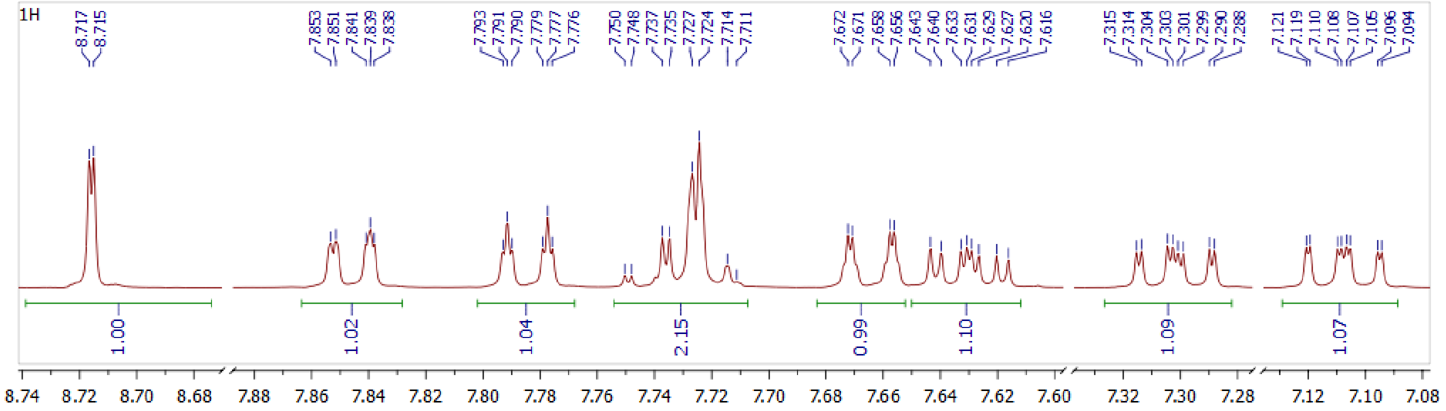

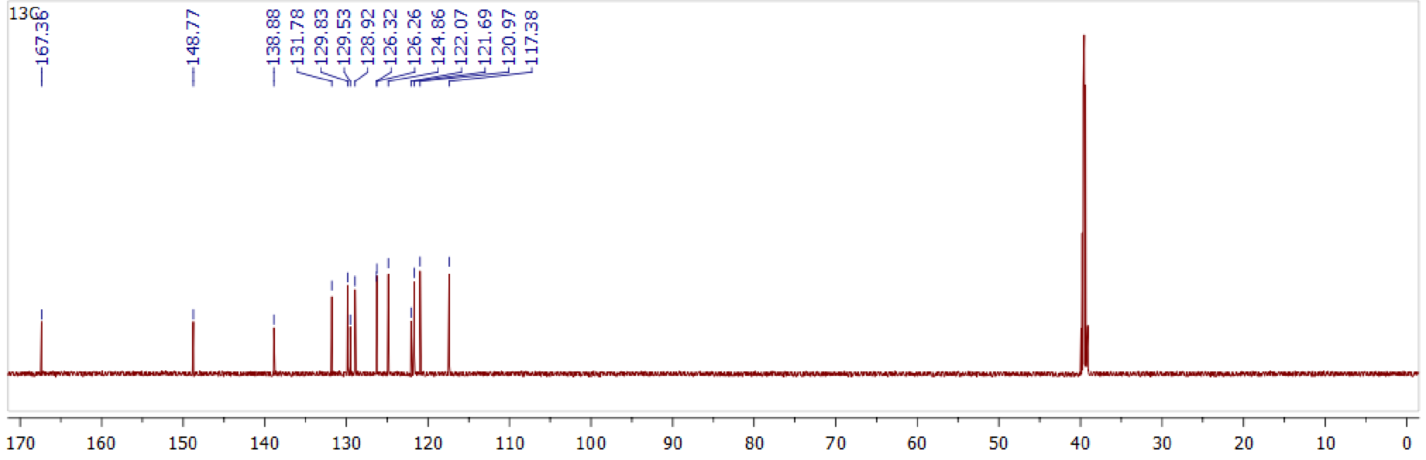

Figure S22. ^{1}H NMR (600 MHz, DMSO-d_6) and ^{13}C NMR (151 MHz, DMSO-d_6) for 2-(2*H*-indazol-2-yl)benzoic acid (**22**).

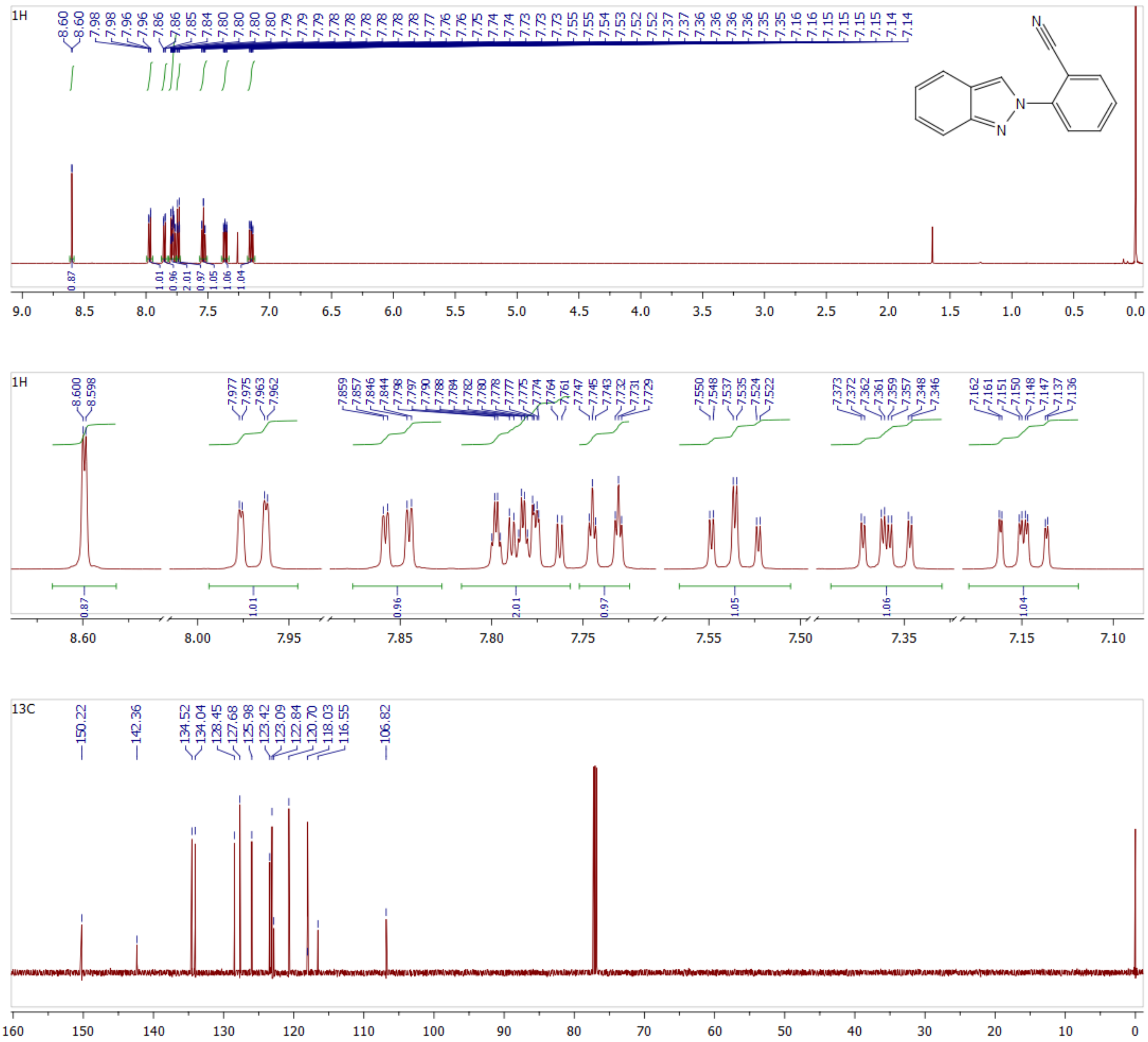

Figure S23. ^{1}H NMR (600 MHz, $CDCl_3$) and ^{13}C NMR (151 MHz, $CDCl_3$) for 2-(2*H*-indazol-2-yl)benzonitrile (**18a**).
